# Supplementary material for: Differential growth enhancement followed by notable microbiota modulation in growing-finishing pigs by Bacillus subtilis strains ps4060, ps4100, and a 50:50 strain mixture
Source: PLoS One. 2024 Sep 9;19(9):e0306014. doi: 10.1371/journal.pone.0306014 (PMC11383229; doi:10.1371/journal.pone.0306014)
Supplement: S1 Fig — (DOCX) [file pone.0306014.s001.docx]

**
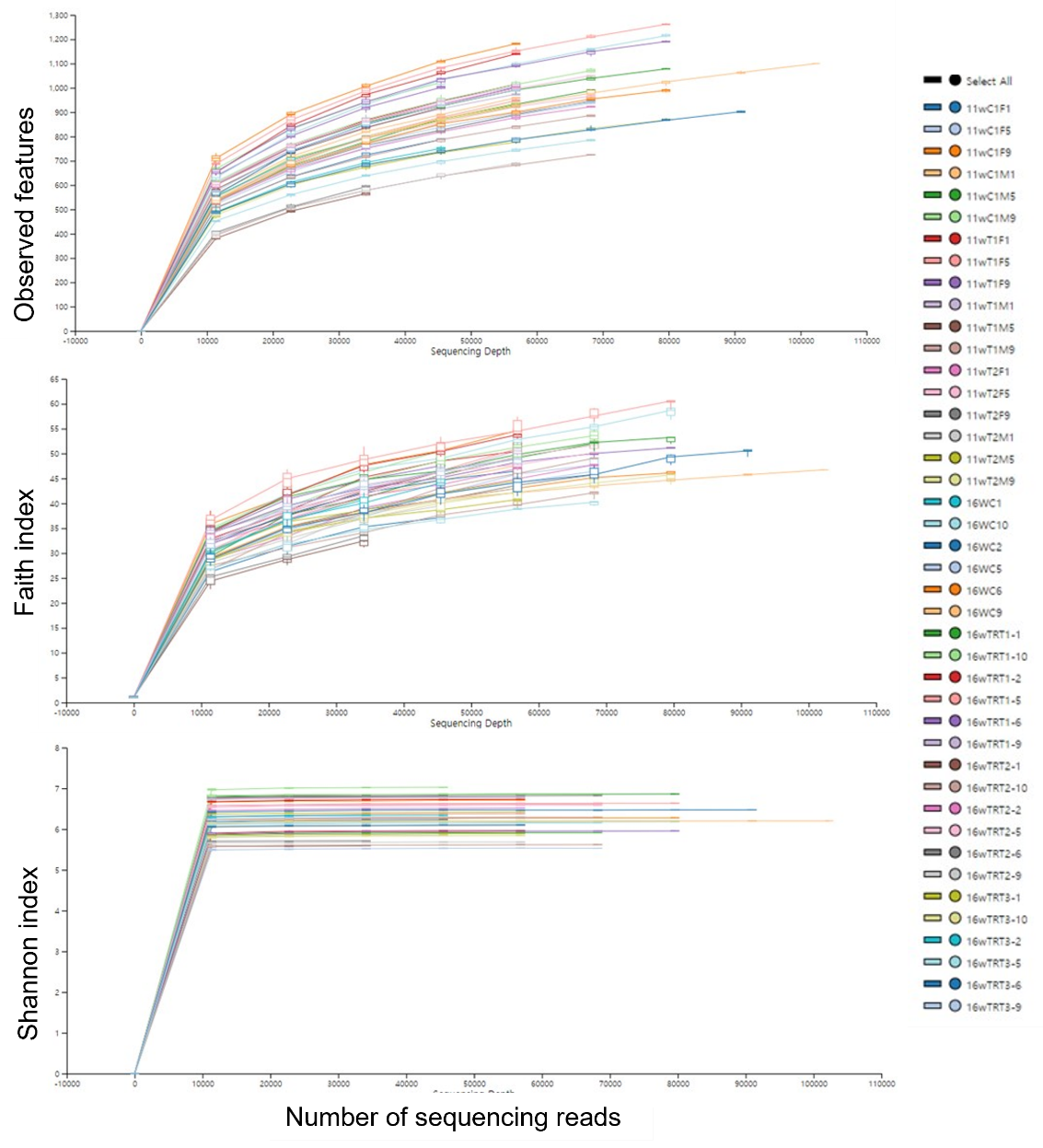
**

**S1. Fig. Rarefaction curves at the genus level showing the diversity of fecal microbiota in growing-finishing pigs supplemented with *Bacillus subtilis* strains ps4100, ps4060, a 50:50 mixture, and a placebo.** The minimum number of sequence reads is 43,609, and the total number of genera is 480 among the 42 samples (week 11 = 18, week 16 = 24). The x-axis represents the library size, and the y-axis represents the corresponding alpha-diversity. Regardless of the choice of diversity measures, the rarefaction curve starts to level out, suggesting that the sequencing depth was sufficient to fully reflect the diversity of the microbiota.
