# Supplementary material for: Differential growth enhancement followed by notable microbiota modulation in growing-finishing pigs by Bacillus subtilis strains ps4060, ps4100, and a 50:50 strain mixture
Source: PLoS One. 2024 Sep 9;19(9):e0306014. doi: 10.1371/journal.pone.0306014 (PMC11383229; doi:10.1371/journal.pone.0306014)
Supplement: S2 Fig — (DOCX) [file pone.0306014.s002.docx]

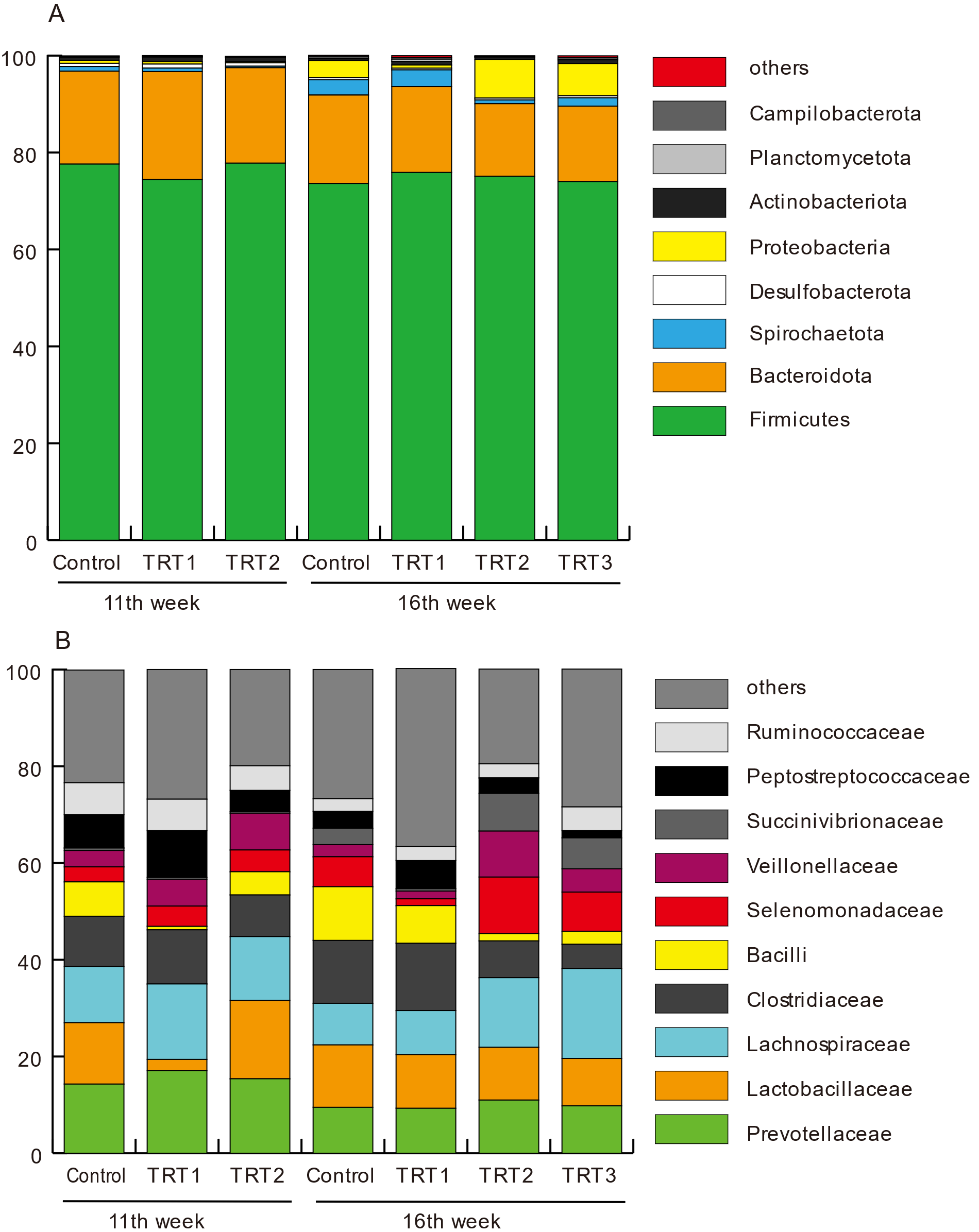


**S2 Fig. Fecal microbiota structure based on 16S rRNA gene analysis.** A. Microbial community structure at the phylum level. B. Microbial community structure at the family level. A total of 11 families—Selenomonadaceae, Oscillospirales__uk, Spirochaetaceae, Oscillospiraceae, Butyricicoccaceae, Anaerovoracaceae, Izemoplasmatales, Oligosphaeraceae, Bacteroidales_RF16_group, UCG.010, and Bifidobacteriaceae—showed differential abundance in ANOVA analysis. All except Selenomonadaceae belong to “others” in the figure.
