# Supplementary material for: Differential growth enhancement followed by notable microbiota modulation in growing-finishing pigs by Bacillus subtilis strains ps4060, ps4100, and a 50:50 strain mixture
Source: PLoS One. 2024 Sep 9;19(9):e0306014. doi: 10.1371/journal.pone.0306014 (PMC11383229; doi:10.1371/journal.pone.0306014)
Supplement: S1 Table — (DOCX) [file pone.0306014.s003.docx]

**S1 Table. Composition of the experimental finishing pig diets (as-fed basis).**

| Composition | Low protein diet (%) |
| --- | --- |
| Corn | 45.06 |
| Wheat | 13 |
| Soybean meal | 23 |
| Rapeseed meal | 2.2 |
| Corn dried distillers  grains with soluble | 5.0 |
| Dicalcium phosphate | 1.06 |
| Limestone | 1 |
| Salt | 0.3 |
| L-Lysine·SO4 (51%) | 0.24 |
| DL-Methionine (50%) | 0.12 |
| L-Tryptophan (10%) | 0.01 |
| L-Threonine (98.5%) | 0.13 |
| Animal fat | 5.3 |
| Molasses | 3.2 |
| Choline (50%) | 0.08 |
| Vitamin premix^a^ | 0.15 |
| Mineral premix^b^ | 0.15 |
| Calculated composition |  |
| Metabolizable energy (kcal·kg−1) | 3400 |
| Lysine (%) | 0.95 |
| Methionine (%) | 0.3 |
| Calciuma (%) | 0.76 |
| Phosphorus (%) | 0.28 |

^a^ Provided per kilogram of complete diet: vitamin A, 10 000 IU; vitamin D3, 2000 IU; vitamin E, 48 IU; vitamin K3, 1.5 mg; riboflavin, 6 mg; niacin, 40 mg; D-pantothenic acid, 17 mg; biotin, 0.2 mg; folic acid, 2 mg; choline, 166 mg; vitamin B6, 2 mg; and vitamin B12, 28 mg.

^b^ Provided per kilogram of complete diet: iron (as FeSO_4_·7H_2_O), 90 mg; copper (as CuSO_4_·5H_2_O), 15 mg; zinc (as ZnSO_4_), 50 mg; manganese (as MnO_2_), 54 mg; iodine (as KI), 0.99 mg; and selenium (as Na_2_SeO_3_·5H_2_O), 0.25 mg.
