# Supplementary material for: Differential growth enhancement followed by notable microbiota modulation in growing-finishing pigs by Bacillus subtilis strains ps4060, ps4100, and a 50:50 strain mixture
Source: PLoS One. 2024 Sep 9;19(9):e0306014. doi: 10.1371/journal.pone.0306014 (PMC11383229; doi:10.1371/journal.pone.0306014)
Supplement: S2 Table — (DOCX) [file pone.0306014.s004.docx]

**S2 Table. Genus-level differential abundance of bacteria among the experimental groups at week 16**

| index | ANCOM | | Con | TRT1 | TRT2 | TRT3 |
| --- | --- | --- | --- | --- | --- | --- |
| g_dgA.11_gut | |  | 88^a^ | 193.5 ^b^ | 18.3 ^c^ | 30.9 ^c^ |
| g_p.1088.a5_gut | |  | 32.9 ^a^ | 205.6 ^b^ | 9.2 ^a^ | 15.2 ^a^ |
| g_Subdoligranulum | |  | 239.6 ^a^ | 106.7 ^b^ | 301 ^a^ | 319.8 ^a^ |
| o_Oscillospirales.__.__ | |  | 6.4 ^a^ | 50.2 ^b^ | 5.6 ^a^ | 16.1 ^a^ |
| g_Family_XIII_AD3011 | |  | 90.8 ^a^ | 272.2 ^b^ | 75.8 ^a^ | 126.9 ^a^ |
| g_UCG.008 | |  | 33.3 ^a^ | 354.7 ^b^ | 15.3 ^a^ | 7.4 ^a^ |
| g_.Eubacterium._nodatum | |  | 13.1 ^a^ | 25.9 ^a^ | 225.8 ^b^ | 154.3 ^b^ |
| g_Lachnospira | |  | 7.7 ^a^ | 23.3 ^a^ | 35.6 ^a^ | 72.9 ^b^ |
| f_Selenomonadaceae | |  | 1572.7 ^a^ | 488.2 ^c^ | 3270.3 ^b^ | 2145.2 ^a^ |
| g_NK4A214 | |  | 734.4 ^a^ | 892.7 ^a^ | 334.4 ^b^ | 829 ^a^ |
| f_Selenomonadaceae.__ | |  | 230.1 ^a^ | 56.2 ^a^ | 540 ^a^ | 1055.5 ^b^ |
| g_Treponema | | y | 1297 ^a^ | 1514.5 ^a^ | 310.5 ^b^ | 739.7 ^b^ |
| g_CAG.352 | |  | 7.9 ^a^ | 11.8 ^a^ | 230.2 ^a^ | 1168.6 ^b^ |
| g_Erysipelotrichaceae_UCG.006 | |  | 8.7 ^a^ | 47.1 ^a^ | 72.3 ^a^ | 340.4 ^b^ |
| g_Anaerovibrio | |  | 216.3 ^a^ | 31.6 ^b^ | 45.7 ^b^ | 25.2 ^b^ |
| g_Izemoplasmatales | | y | 4.8 ^a^ | 58.9 ^b^ | 6.9 ^a^ | 47.7 ^b^ |
| g_Alloprevotella | |  | 98.8 ^a^ | 249.5 ^b^ | 69.7 ^a^ | 69.8 ^a^ |
| g_Shuttleworthia | |  | 478.5 ^a^ | 355.7 ^a^ | 3933.8 ^b^ | 5730.1 ^b^ |
| g_Bacteroidales_RF16 | | y | 1061.4 ^a^ | 135.6 ^b^ | 45.6 ^b^ | 63.6 ^b^ |
| g_UCG.002 | |  | 399.7 ^a^ | 741 ^b^ | 247.5 ^a^ | 470.7 ^a^ |
| g_UCG.010 | |  | 273.9 ^a^ | 598.7 ^b^ | 138.7 ^a^ | 557.3 ^b^ |
| g_WPS.2 | | y | 0.7 ^a^ | 11.9 ^a^ | 12.6 ^a^ | 70.3 ^b^ |
| f_Prevotellaceae.__ | |  | 88.3 ^a^ | 272.4 ^a^ | 389.1 ^b^ | 644.3 ^b^ |
| g_Bifidobacterium | | y | 4.7 ^a^ | 0.4 ^a^ | 82.5 ^b^ | 74.5 ^b^ |
| g_Monoglobus | |  | 6.7 ^a^ | 31.6 ^b^ | 9.1 ^a^ | 32 ^b^ |
| g_Sphaerochaeta | | y | 138.7 ^a^ | 51 ^b^ | 34.1 ^b^ | 30.9 ^b^ |
| g_Succinivibrio | | y | 1537.3 ^a^ | 126.9 ^b^ | 597.5 ^b^ | 238.4 ^b^ |
| g_UCG.004 | |  | 420.3 ^a^ | 159.5 ^b^ | 270.8 ^ab^ | 475.3 ^a^ |
| g_p.2534.18B5_gut | |  | 48.7 ^a^ | 252.4 ^b^ | 86 ^a^ | 6 ^a^ |
| g_Blautia | |  | 350 ^a^ | 179.2 ^b^ | 159 ^b^ | 97.8 ^b^ |
| g_Christensenellaceae_R.7 | |  | 265.1 ^a^ | 1355.6 ^b^ | 283.8 ^a^ | 637.7 ^a^ |
| g_Bacteroides | | y | 16.5 ^a^ | 68.7 ^b^ | 1.8 ^a^ | 0.8 ^a^ |
| g_Selenomonas | | y | 741.2 ^a^ | 45.8 ^b^ | 1203.6 ^a^ | 399 ^ab^ |
| g_Faecalibacterium | | y | 184.9 ^a^ | 50.4 ^b^ | 129.5 ^a^ | 30.4 ^b^ |
| p_Firmicutes.__.__.__.__ | |  | 0.8 ^a^ | 7.6 ^a^ | 38.5 ^b^ | 37.9 ^b^ |
| g_Lachnospiraceae_NK4A136 | | y | 29.3 ^a^ | 135.2 ^b^ | 28 ^a^ | 4.9 ^a^ |
| g_Prevotellaceae_NK3B31 | |  | 637.3 ^a^ | 617.4 ^a^ | 148.6 ^b^ | 193.5 ^b^ |
| g_Oribacterium | |  | 160.5 ^a^ | 70.8 ^a^ | 788.2 ^b^ | 418.8 ^ab^ |
| g_Rikenellaceae_RC9_gut | |  | 1216.8 ^a^ | 1358.1 ^a^ | 596.3 ^b^ | 959.1 ^ab^ |
| g_Z20 | |  | 10.2 ^a^ | 32.4 ^ab^ | 0.3 ^a^ | 36.9 ^b^ |
| g_Erysipelotrichaceae_UCG.009 | |  | 0 ^a^ | 25.4 ^a^ | 49.1 ^ab^ | 87.5 ^b^ |
| f_Prevotellaceae | |  | 495.4 ^a^ | 28.2 ^b^ | 142.9 ^b^ | 138.2 ^b^ |
| g_Clostridia_vadinBB60 | | y | 232.7 ^a^ | 446.3 ^b^ | 139.2 ^a^ | 218 ^a^ |
| f_Oscillospiraceae.__ | |  | 98.9 ^a^ | 262.7 ^b^ | 91 ^a^ | 119.1 ^a^ |
| g_UCG.005 | |  | 791.5 ^a^ | 1117.7 ^a^ | 310.4 ^b^ | 878.7 ^a^ |
| g_Mitsuokella | |  | 10.6 ^a^ | 11 ^a^ | 273.4 ^b^ | 44.2 ^a^ |
| g_Fusicatenibacter | |  | 86.4 ^a^ | 18.9 ^b^ | 20.3 ^b^ | 6.6 ^b^ |
| g_.Eubacterium._coprostanoligenes | |  | 282.5 ^a^ | 570.2 ^ab^ | 336.5 ^a^ | 625.1 ^b^ |
| g_Agathobacter | | y | 348.7 ^a^ | 5.2 ^b^ | 26.6 ^b^ | 45.2 ^b^ |
| g_Clostridia_UCG.014 | |  | 53.2 ^a^ | 239.3 ^b^ | 120.9 ^a^ | 95.9 ^a^ |
| f_Oscillospiraceae | |  | 61.4 ^a^ | 64.3 ^a^ | 0 ^b^ | 5.1 ^b^ |
| f_Lachnospiraceae.__ | |  | 905.7 ^a^ | 744 ^ab^ | 324.1 ^b^ | 422 ^b^ |
| g_Parabacteroides | |  | 246.1 ^a^ | 299.7 ^a^ | 69.4 ^b^ | 104.7 ^ab^ |
| g_Succinivibrionaceae_UCG.001 | |  | 20.3 ^a^ | 89.1 ^a^ | 2916.9 ^b^ | 2652.9 ^b^ |
| g_Fournierella | |  | 48.8 ^a^ | 21.3 ^b^ | 11.5 ^b^ | 13.8 ^b^ |
| f_Ruminococcaceae | |  | 65.5 ^a^ | 171.2 ^b^ | 62.3 ^a^ | 64 ^a^ |
| g_CAG.873 | |  | 1.6 ^a^ | 35.4 ^a^ | 29.2 ^ab^ | 77.9 ^b^ |
| g_Candidatus_Soleaferrea | |  | 26.7 ^a^ | 61.7 ^b^ | 25.7 ^a^ | 21.2 ^a^ |
| g_Prevotellaceae_UCG.001 | |  | 229.9 ^a^ | 689.3 ^b^ | 84.8 ^a^ | 214.4 ^a^ |
| g_Lachnospiraceae_NK4B4 | |  | 28 ^a^ | 80.3 ^b^ | 16.1 ^a^ | 52.9 ^a^ |
| g_Dialister | |  | 185.8 ^a^ | 184.4 ^a^ | 858.3 ^b^ | 518.2 ^ab^ |
| o_Bacteroidales.__.__ | |  | 27.9 ^a^ | 54.2 ^ab^ | 9.3 ^a^ | 82.1 ^b^ |
| g_Marvinbryantia | |  | 122.6 ^a^ | 81.2 ^b^ | 63 ^b^ | 47.1 ^b^ |
| g_Helicobacter | |  | 43.1 ^a^ | 25.5 ^ab^ | 9 ^b^ | 17.9 ^ab^ |
| g_Lachnospiraceae_XPB1014_group | | y | 202.6 ^ab^ | 322 ^a^ | 114.3 ^ab^ | 30.2 ^b^ |
| g_Syntrophococcus * | | y | 0 ^a^ | 275.2 ^ab^ | 153.4 ^ab^ | 356.9 ^b^ |
| g_.Ruminococcus._gauvreauii | |  | 100.7 ^a^ | 553.2 ^ab^ | 167.7 ^ab^ | 630.3 ^b^ |
| g_Coprococcus * | |  | 378.8 ^a^ | 426.7 ^a^ | 195 ^ab^ | 77.7 ^b^ |
| g_Lachnospiraceae_AC2044 * | | y | 26 ^ab^ | 52.6 ^b^ | 10.6 ^a^ | 7.6 ^a^ |
| f_Paludibacteraceae | |  | 80.5 ^ab^ | 578.4 ^b^ | 5.2 ^a^ | 93.3 ^ab^ |
| g_Ruminococcus | |  | 353.2 ^ab^ | 581.6 ^b^ | 254.4 ^a^ | 345.4 ^a^ |
| g_Clostridium_sensu_stricto_1 | |  | 5804.3 ^b^ | 6162 ^b^ | 3408.3 ^ab^ | 2210.9 ^a^ |
| g_Asteroleplasma * | | y | 70.4 ^ab^ | 39.1 ^a^ | 233.2 ^b^ | 181.4 ^ab^ |
| g_Dorea | |  | 91.2 ^a^ | 49.9 ^ab^ | 29.7 ^b^ | 28.4 ^b^ |
| g_Prevotellaceae_UCG.004 * | |  | 34.9 ^a^ | 72.3 ^ab^ | 48.7 ^a^ | 121.3 ^b^ |
| g_Fibrobacter | |  | 22.3 ^ab^ | 43.4 ^a^ | 8.5 ^b^ | 41.6 ^ab^ |
| g_Olsenella * | |  | 13.9 ^a^ | 182.2 ^b^ | 69 ^ab^ | 174.2 ^b^ |
| g_Catenibacterium | |  | 88.9 ^a^ | 310.5 ^b^ | 116.3 ^a^ | 45.5 ^a^ |
| g_Lachnospiraceae_ND3007_group* | | y | 40.8 ^ab^ | 68.1 ^ab^ | 36.2 ^b^ | 12.7 ^a^ |
| g_Lachnospiraceae_FCS020_group | |  | 33.3 ^ab^ | 42.4 ^ab^ | 28.4 ^b^ | 14.4 ^a^ |
| g_Sutterella | |  | 39.2 ^ab^ | 19.1 ^b^ | 40.2 ^ab^ | 59.7 ^a^ |
| g_Phascolarctobacterium | |  | 358.9 ^a^ | 196 ^ab^ | 88 ^b^ | 119.3 ^ab^ |
| g_Oscillibacter | |  | 55.4 ^ab^ | 95.8 ^b^ | 52.3 ^ab^ | 32.3 ^a^ |
| g_Eubacterium_hallii * | |  | 155.4 ^ab^ | 201.2 ^b^ | 122.7 ^ab^ | 138.4 ^a^ |
| c_Bacilli.__.__.__ | |  | 5043.8 ^a^ | 3541.1 ^a^ | 678.8 ^b^ | 1218.1 ^b^ |
| g_Acidaminococcus | | y | 29.9 ^a^ | 58.3 ^a^ | 356.7 ^b^ | 437 ^b^ |
| g_.Eubacterium._siraeum_group | |  | 29.8 ^a^ | 86.4 ^b^ | 23.7 ^a^ | 18.1 ^a^ |
| f_uncultured | |  | 10.9 ^a^ | 16.7 ^a^ | 56.8 ^a^ | 130.6 ^b^ |
| g_Prevotellaceae_UCG.003 | |  | 253.5 ^a^ | 241 ^a^ | 62.4 ^b^ | 62.3 ^b^ |
| g_Terrisporobacter | |  | 1008.8 ^ab^ | 1847.5 ^a^ | 748.5 ^ab^ | 411.8 ^b^ |
| g_Megasphaera | |  | 932.3 ^a^ | 541.5 ^a^ | 3426.9 ^b^ | 1636.2 ^ab^ |
| g_Clostridium_sensu_stricto_6 | |  | 105.1 ^ab^ | 138.9 ^b^ | 53.1 ^a^ | 55.9 ^a^ |
| g_Peptococcus | | y | 44.3 ^a^ | 34.4 ^a^ | 14.3 ^b^ | 13.5 ^b^ |
| g_Intestinibacter * | |  | 206.7 ^ab^ | 151.5 ^ab^ | 131.2 ^b^ | 46.4 ^a^ |
| g_Colidextribacter | |  | 61.5 ^ab^ | 53.2 ^ab^ | 49.9 ^a^ | 84 ^b^ |
| g_Prevotella | |  | 2475 ^a^ | 2058 ^a^ | 4044.9 ^b^ | 2988.1 ^a^ |

A total of 96 taxa were selected by ANOVA (*p* < 0.008). Distinct superscripts denote significant differences among the means within the same row as determined by Tukey’s tests. Seven taxa with an asterisk (*) indicate significance at the level of 0.05 < *p* < 0.008.
