# Supplementary material for: Differential growth enhancement followed by notable microbiota modulation in growing-finishing pigs by Bacillus subtilis strains ps4060, ps4100, and a 50:50 strain mixture
Source: PLoS One. 2024 Sep 9;19(9):e0306014. doi: 10.1371/journal.pone.0306014 (PMC11383229; doi:10.1371/journal.pone.0306014)
Supplement: S1 Appendix — (DOCX) [file pone.0306014.s005.docx]

**S1 Appendix**

**Digestibility, Carcass grade, and meat quality**

**MATERIAL AND METHODS**

**Digestibility**

Chromium oxide (0.2%) was introduced into the diet as an indigestible marker seven days prior to fecal collection at the end of weeks 6 and 16. This addition facilitated the assessment of dry matter, nitrogen, and energy digestibility in accordance with the Association of Official Analytical Chemists International (AOAC) guidelines [1]. This method was previously described [2]. Fecal samples from at least two pigs per pen (one barrow and one gilt) were randomly collected, combined, and stored at -20°C. A representative sample underwent a drying process at 70°C for 72 hours, followed by grinding and sieving through a 1 mm-screen with feed samples. The digestibility of dry matter and nitrogen was analyzed following AOAC (2006) guidelines (method 935.29 for dry matter and method 990.03 for nitrogen). UV spectrophotometry was used for chromium quantification (Shimadzu, UV-1201, Kyoto, Japan). The nutrient digestibility equation is as follows:

ND (%) = 100 - [(NF/ND) × (CrD/CrF)] × 100,

Here, ND represents nutrient digestibility, and NF, ND, CrD, and CrF represent the nutrient concentration in the fecal sample, nutrient concentration in the diet, chromium concentration in the diet, and chromium concentration in the fecal sample, respectively. To determine gross energy (GE), a 2 g (ranging from 1.9980 to 2.0020) fecal sample was placed inside a Parr 6400 oxygen calorimeter (Parr instrument, Moline, IL, USA) and run for approximately 7 minutes. The heat combustion within the samples was recorded for statistical analysis. The same procedure was applied to the feed samples. The final calculation for GE was the energy in feed minus the energy in feces.

**Carcass grade and meat quality**

The carcasses were stored at 4°C in a conventional chiller. Lean and fat meat samples were obtained from loin cuts. Back fat thickness was measured at three sites on the pigs' bodies using a real-time ultrasound instrument (Piglog 105, SFK Technology). The color attributes of the meat samples (L*, a*, b*) were measured using a Minolta CR-410 chroma meter (Konica Minolta Sensing, Inc., Osaka, Japan). In this evaluation system, each character represents the lightness, position on the red-green axis, and position on the yellow-blue axis, enabling objective measurement and comparison of meat color. This helps assess freshness, quality, and potential issues such as spoilage or discoloration. Sensory evaluation scores for color, marbling, and firmness followed National Pork Producers Council standards. Drip loss was assessed using approximately 4 g of meat via the plastic bag method [3], while cooking loss was calculated as the ratio of cooked weight to uncooked weight. pH values were measured in duplicate for each sample using a glass-electrode pH meter (WTW pH 340-A, WTH Measurement Systems, Inc., Ft. Myers, FL, USA). The water holding capacity (WHC) was determined following established guidelines [4]. The longissimus muscle area (LMA) was measured by tracing the muscle surface at the 10th rib. The meat grade was determined according to the Korea Institute for Animal Products Quality Evaluation (KAPE, 2010) standards, with the carcass backfat thickness adjusted to a live weight of 115 kg, as previously described [5].

**Results**

**Diet intake and nutrient digestibility**

There was no difference in average daily feed intake (ADFI), regardless of the increase in ADG. A positive trend for G:F was discernible among the *Bacillus*-fed groups although a limited sample size hindered statistical significance. Additionally, there were no differences in fecal dry matter, nitrogen, or digestible energy (Table 1). These feed and fecal data suggested that *Bacillus* supplementation did not affect dietary intake or nutrient digestion.

**Table 1. The effect of dietary supplementation with *Bacillus subtilis* on nutrient digestibility**

| Items | CON | TRT1 | TRT2 | TRT3 | SEM | *P* - value |
| --- | --- | --- | --- | --- | --- | --- |
| Week 6 |  |  |  |  |  |  |
| Dry matter | 76.31 | 77.03 | 77.34 | 77.45 | 0.19 | 0.1555 |
| Nitrogen | 77.15 | 77.00 | 75.01 | 75.12 | 1.06 | 0.8386 |
| Digestible energy | 76.55 | 75.29 | 76.42 | 75.85 | 0.83 | 0.9539 |
| Week 16 |  |  |  |  |  |  |
| Dry matter | 70.10 | 72.03 | 69.34 | 69.86 | 0.75 | 0.6310 |
| Nitrogen | 67.95 | 68.91 | 67.28 | 69.72 | 0.82 | 0.7509 |
| Digestible energy | 68.71 | 68.76 | 68.51 | 70.82 | 0.82 | 0.7420 |

Abbreviations: CON, basal diet; TRT1, basal diet + ps4100; TRT2, basal diet + ps4060; TRT3, basal diet + 50:50 mixture of ps4100 and ps4060; SEM, standard error of means.

**Carcass grade and meat quality**

The number of carcasses with the highest grade (1+) under the grading system in the Republic of Korea increased among the three experimental groups compared to the control group (Table 2). Specifically, there were nine, ten, and ten highest-grade carcasses (1+) out of 40 pigs in the ps4100, ps4060, and 50:50 mixture groups, respectively, compared to eight out of 40 pigs in the control group. In addition, the desirable color of the meat, particularly redness, was significantly greater in the ps4060 and 50:50 mixture groups than in the control and ps4100 groups (*p* < 0.01) (Table 3). The enhanced redness surpassed the less desirable greenness among the assessed color characteristics of pork meat.

**Table 2. The effect of dietary supplementation with *Bacillus subtilis* on carcass grade**

| Items | CON | TRT1 | TRT2 | TRT3 | SEM | *P* - value |
| --- | --- | --- | --- | --- | --- | --- |
| Carcass weight, kg | 90.45 | 90.90 | 91.28 | 91.13 | 0.36 | 0.8632 |
| Backfat thickness, mm | 18.25 | 18.30 | 18.48 | 18.40 | 0.21 | 0.9813 |
| 1+, % | 8 | 9 | 10 | 10 | - | - |
| 1, % | 16 | 14 | 16 | 15 | - | - |
| 2, % | 16 | 17 | 14 | 15 | - | - |

Abbreviation: CON, basal diet; TRT1, Basal Diet + ps4100; TRT2, Basal diet + ps4060; TRT3, Basal diet + 50:50 mixture of ps4100 and ps4060; SEM, Standard error of means.

**Table 3. The effect of dietary supplementation with *Bacillus subtilis* on meat quality**

| Items | CON | TRT1 | TRT2 | TRT3 | SEM^2^ | *P* - value^3^ |
| --- | --- | --- | --- | --- | --- | --- |
| pH | 5.15 | 5.22 | 5.30 | 5.29 | 0.03 | 0.2778 |
| Longissimus muscle area, mm^2^ | 7274.79 | 7365.73 | 7567.85 | 7124.91 | 137.71 | 0.7571 |
| Water holding capacity, % | 45.91 | 45.16 | 44.93 | 50.60 | 1.49 | 0.5378 |
| Cooking loss, % | 27.24 | 27.11 | 27.84 | 29.73 | 1.29 | 0.9045 |
| Drip loss, % |  |  |  |  |  |  |
| d1 | 7.40 | 8.25 | 7.59 | 7.38 | 0.24 | 0.5677 |
| d3 | 14.48 | 13.63 | 14.08 | 13.88 | 0.35 | 0.8736 |
| d5 | 19.54 | 19.56 | 19.34 | 19.81 | 0.09 | 0.4059 |
| d7 | 24.86 | 24.69 | 24.48 | 24.50 | 0.07 | 0.1172 |
| Meat color |  |  |  |  |  |  |
| L* | 52.27 | 51.57 | 51.83 | 51.67 | 0.13 | 0.2724 |
| a* | 14.18^b^ | 14.19^b^ | 14.57^a^ | 14.57^a^ | 0.06 | 0.0068 |
| b* | 6.36 | 6.36 | 6.21 | 6.55 | 0.05 | 0.1052 |
| Sensory evaluation |  |  |  |  |  |  |
| Color | 3.19 | 3.28 | 3.22 | 3.19 | 0.05 | 0.9245 |
| Marbling | 3.25 | 3.16 | 3.13 | 3.25 | 0.07 | 0.8972 |
| Firmness | 3.22 | 3.13 | 3.25 | 3.31 | 0.04 | 0.5067 |

Abbreviations: CON, basal diet; TRT1, basal diet + ps4100; TRT2, basal diet + ps4060; TRT3, basal diet + 50:50 mixture of ps4100 and ps4060; SEM, standard error of means. "L," "a," and "b" represent the evaluation of lightness, the spectrum from green to red, and the spectrum from blue to yellow, respectively, of meat colors.

**References**

1. USDA. Offficial Methods of Analysis of the Association of Official Analytical Chemists International. Gaithersburg, MD: Association of Official Agricultural Chemists International; 2006.

2. Lan R, Kim IH. Effects of *Bacillus licheniformis* and *Bacillus subtilis* complex on growth performance and faecal noxious gas emissions in growing-finishing pigs. Journal of the science of food and agriculture. 2019;99(4):1554-60. doi: 10.1002/jsfa.9333. PubMed PMID: 30144078.

3. Honikel KO. Reference methods for the assessment of physical characteristics of meat. Meat science. 1998;49(4):447-57. doi: 10.1016/s0309-1740(98)00034-5. PubMed PMID: 22060626.

4. Kauffman RG, Eikelenboom G, van der Wal PG, Engel B, Zaar M. A comparison of methods to estimate water-holding capacity in post-rigor porcine muscle. Meat science. 1986;18(4):307-22. doi: 10.1016/0309-1740(86)90020-3. PubMed PMID: 22055735.

5. Ha DM, Kim GD, Han JC, Jeong JY, Park MJ, Park BC, et al. Effects of dietary energy level on growth efficiency and carcass quality traits of finishing pigs. J Anim Sci Technol. 2010;52(191-198).
