# Supplementary material for: Differential growth enhancement followed by notable microbiota modulation in growing-finishing pigs by Bacillus subtilis strains ps4060, ps4100, and a 50:50 strain mixture
Source: PLoS One. 2024 Sep 9;19(9):e0306014. doi: 10.1371/journal.pone.0306014 (PMC11383229; doi:10.1371/journal.pone.0306014)
Supplement: S2 Appendix — (DOCX) [file pone.0306014.s006.docx]

Raw data for Table 1

| **TRT** | **REP** | **PEN** | **PIG No** | **Body Weight** | | | | **ADG1**  **(week6)** | **ADG2**  **(week 11)** | **ADG3**  **(week 16)** | **TADG** | cumulative ADG |
| --- | --- | --- | --- | --- | --- | --- | --- | --- | --- | --- | --- | --- |
|  |  |  |  | **Initial** | **Week 6** | **Week 11** | **Week 16** |  |  |  |  | **Week 11** |
| **Con** | **1** | **1** | **258** | 29.48 | 57.99 | 87.12 | 118.59 | 679 | 832 | 899 | 796 | **748.6** |
| **Con** | **1** | **1** | **285** | 28.76 | 59.64 | 89.13 | 124.88 | 735 | 843 | 1021 | 858 | **784.0** |
| **Con** | **1** | **1** | **662** | 29.12 | 59.53 | 89.15 | 124.15 | 724 | 846 | 1000 | 848 | **779.6** |
| **Con** | **1** | **1** | **678** | 29.14 | 56.91 | 83.15 | 118.56 | 661 | 750 | 1012 | 798 | **701.4** |
| **Con** | **2** | **10** | **239** | 27.57 | 54.91 | 80.95 | 116.48 | 651 | 744 | 1015 | 794 | **693.2** |
| **Con** | **2** | **10** | **623** | 27.94 | 53.73 | 79.88 | 113.80 | 614 | 747 | 969 | 767 | **674.5** |
| **Con** | **2** | **10** | **635** | 28.03 | 53.06 | 77.82 | 110.84 | 596 | 707 | 943 | 739 | **646.6** |
| **Con** | **2** | **10** | **686** | 28.66 | 55.16 | 80.61 | 112.98 | 631 | 727 | 925 | 753 | **674.7** |
| **Con** | **3** | **14** | **257** | 26.78 | 57.01 | 86.46 | 119.46 | 720 | 841 | 943 | 828 | **775.1** |
| **Con** | **3** | **14** | **640** | 27.56 | 57.42 | 85.50 | 118.97 | 711 | 802 | 956 | 816 | **752.5** |
| **Con** | **3** | **14** | **681** | 26.93 | 57.97 | 87.77 | 121.81 | 739 | 851 | 973 | 847 | **790.1** |
| **Con** | **3** | **14** | **683** | 26.97 | 59.24 | 89.68 | 122.74 | 768 | 870 | 945 | 855 | **814.4** |
| **Con** | **4** | **18** | **211** | 26.12 | 56.86 | 86.18 | 119.46 | 732 | 838 | 951 | 833 | **780.0** |
| **Con** | **4** | **18** | **280** | 26.61 | 58.00 | 87.10 | 119.41 | 747 | 831 | 923 | 829 | **785.6** |
| **Con** | **4** | **18** | **647** | 25.85 | 54.35 | 81.96 | 113.41 | 679 | 789 | 899 | 782 | **728.7** |
| **Con** | **4** | **18** | **688** | 26.07 | 55.72 | 84.02 | 118.32 | 706 | 809 | 980 | 824 | **752.6** |
| **Con** | **5** | **22** | **220** | 25.34 | 54.32 | 81.48 | 110.06 | 690 | 776 | 817 | 756 | **729.1** |
| **Con** | **5** | **22** | **267** | 25.34 | 56.65 | 86.15 | 115.15 | 745 | 843 | 829 | 802 | **789.7** |
| **Con** | **5** | **22** | **603** | 24.83 | 53.15 | 80.95 | 109.12 | 674 | 794 | 805 | 753 | **728.8** |
| **Con** | **5** | **22** | **692** | 25.78 | 53.92 | 81.11 | 109.89 | 670 | 777 | 822 | 751 | **718.6** |
| **Con** | **6** | **26** | **227** | 23.83 | 51.93 | 79.77 | 110.54 | 669 | 795 | 879 | 774 | **726.5** |
| **Con** | **6** | **26** | **255** | 24.80 | 55.18 | 84.95 | 117.20 | 723 | 851 | 921 | 825 | **781.2** |
| **Con** | **6** | **26** | **271** | 24.25 | 54.03 | 82.82 | 115.78 | 709 | 823 | 942 | 817 | **760.6** |
| **Con** | **6** | **26** | **609** | 24.27 | 52.01 | 78.88 | 109.75 | 660 | 768 | 882 | 763 | **709.2** |
| **Con** | **7** | **35** | **269** | 23.36 | 49.73 | 76.23 | 107.44 | 628 | 757 | 892 | 751 | **686.6** |
| **Con** | **7** | **35** | **629** | 23.78 | 49.99 | 76.66 | 107.73 | 624 | 762 | 888 | 750 | **686.8** |
| **Con** | **7** | **35** | **661** | 23.24 | 53.10 | 81.55 | 113.85 | 711 | 813 | 923 | 809 | **757.3** |
| **Con** | **7** | **35** | **664** | 22.79 | 51.99 | 80.17 | 111.89 | 695 | 805 | 906 | 796 | **745.2** |
| **Con** | **8** | **39** | **263** | 22.30 | 51.07 | 78.79 | 109.56 | 685 | 792 | 879 | 779 | **733.6** |
| **Con** | **8** | **39** | **639** | 22.34 | 53.52 | 83.97 | 111.86 | 742 | 870 | 797 | 799 | **800.4** |
| **Con** | **8** | **39** | **676** | 21.91 | 52.57 | 83.85 | 113.73 | 730 | 894 | 854 | 820 | **804.4** |
| **Con** | **8** | **39** | **680** | 22.79 | 51.71 | 79.55 | 110.85 | 689 | 795 | 894 | 786 | **737.1** |
| **Con** | **9** | **43** | **254** | 21.47 | 52.14 | 81.61 | 113.51 | 730 | 842 | 911 | 822 | **781.0** |
| **Con** | **9** | **43** | **612** | 21.09 | 48.35 | 74.89 | 106.42 | 649 | 758 | 901 | 762 | **698.7** |
| **Con** | **9** | **43** | **627** | 21.89 | 50.35 | 78.96 | 109.29 | 678 | 817 | 867 | 780 | **741.2** |
| **Con** | **9** | **43** | **667** | 21.47 | 50.74 | 78.95 | 109.25 | 697 | 806 | 866 | 784 | **746.5** |
| **Con** | **10** | **47** | **204** | 20.22 | 46.71 | 72.94 | 105.94 | 631 | 749 | 943 | 765 | **684.7** |
| **Con** | **10** | **47** | **228** | 21.07 | 50.22 | 76.13 | 107.73 | 694 | 740 | 903 | 774 | **715.1** |
| **Con** | **10** | **47** | **628** | 20.64 | 50.52 | 78.91 | 110.85 | 711 | 811 | 913 | 805 | **756.8** |
| **Con** | **10** | **47** | **677** | 20.69 | 47.68 | 73.55 | 106.94 | 643 | 739 | 954 | 770 | **686.5** |
| **TRT1** | **1** | **2** | **279** | 29.47 | 56.22 | 81.72 | 115.95 | 637 | 729 | 978 | 772 | **678.6** |
| **TRT1** | **1** | **2** | **286** | 28.91 | 57.57 | 84.63 | 118.45 | 682 | 773 | 966 | 799 | **723.6** |
| **TRT1** | **1** | **2** | **610** | 29.15 | 57.51 | 83.78 | 116.86 | 675 | 751 | 945 | 783 | **709.5** |
| **TRT1** | **1** | **2** | **637** | 28.76 | 55.33 | 80.49 | 114.95 | 633 | 719 | 985 | 770 | **671.8** |
| **TRT1** | **2** | **6** | **237** | 28.64 | 55.09 | 83.95 | 115.44 | 630 | 825 | 900 | 775 | **718.3** |
| **TRT1** | **2** | **6** | **277** | 27.63 | 53.88 | 82.46 | 114.63 | 625 | 817 | 919 | 777 | **712.1** |
| **TRT1** | **2** | **6** | **283** | 28.07 | 57.36 | 87.62 | 119.44 | 697 | 865 | 909 | 816 | **773.4** |
| **TRT1** | **2** | **6** | **614** | 27.89 | 53.81 | 79.11 | 111.96 | 617 | 723 | 939 | 751 | **665.2** |
| **TRT1** | **3** | **15** | **223** | 26.80 | 60.91 | 92.15 | 126.19 | 812 | 893 | 973 | 887 | **848.7** |
| **TRT1** | **3** | **15** | **240** | 26.91 | 60.34 | 91.95 | 125.71 | 796 | 903 | 965 | 882 | **844.7** |
| **TRT1** | **3** | **15** | **638** | 27.07 | 61.76 | 90.68 | 124.71 | 826 | 826 | 972 | 872 | **826.1** |
| **TRT1** | **3** | **15** | **650** | 27.41 | 58.28 | 87.13 | 122.65 | 735 | 824 | 1015 | 850 | **775.6** |
| **TRT1** | **4** | **19** | **244** | 25.86 | 55.38 | 83.92 | 115.41 | 703 | 815 | 900 | 800 | **754.0** |
| **TRT1** | **4** | **19** | **248** | 26.59 | 59.36 | 89.43 | 122.11 | 780 | 859 | 934 | 853 | **816.1** |
| **TRT1** | **4** | **19** | **291** | 26.23 | 56.30 | 85.49 | 117.56 | 716 | 834 | 916 | 815 | **769.6** |
| **TRT1** | **4** | **19** | **653** | 26.03 | 59.05 | 90.52 | 123.82 | 786 | 899 | 951 | 873 | **837.5** |
| **TRT1** | **5** | **23** | **250** | 25.30 | 52.27 | 78.19 | 109.88 | 642 | 741 | 905 | 755 | **686.9** |
| **TRT1** | **5** | **23** | **264** | 25.76 | 56.72 | 84.98 | 115.69 | 737 | 807 | 877 | 803 | **769.1** |
| **TRT1** | **5** | **23** | **601** | 24.88 | 52.52 | 80.15 | 108.66 | 658 | 789 | 815 | 748 | **717.8** |
| **TRT1** | **5** | **23** | **684** | 25.47 | 53.28 | 79.93 | 110.69 | 662 | 761 | 879 | 761 | **707.3** |
| **TRT1** | **6** | **27** | **212** | 24.36 | 52.79 | 80.67 | 111.22 | 677 | 797 | 873 | 776 | **731.3** |
| **TRT1** | **6** | **27** | **274** | 24.77 | 56.44 | 86.90 | 115.06 | 754 | 870 | 805 | 806 | **806.9** |
| **TRT1** | **6** | **27** | **620** | 23.84 | 52.14 | 79.36 | 109.45 | 674 | 778 | 860 | 764 | **721.0** |
| **TRT1** | **6** | **27** | **622** | 24.24 | 55.74 | 85.72 | 114.41 | 750 | 857 | 820 | 805 | **798.4** |
| **TRT1** | **7** | **31** | **266** | 22.80 | 53.84 | 83.87 | 117.68 | 739 | 858 | 966 | 847 | **793.1** |
| **TRT1** | **7** | **31** | **287** | 23.69 | 53.81 | 81.85 | 115.91 | 717 | 801 | 973 | 823 | **755.3** |
| **TRT1** | **7** | **31** | **615** | 23.15 | 50.24 | 75.88 | 110.89 | 645 | 733 | 1000 | 783 | **684.8** |
| **TRT1** | **7** | **31** | **685** | 23.40 | 53.60 | 81.78 | 115.84 | 719 | 805 | 973 | 825 | **758.2** |
| **TRT1** | **8** | **40** | **208** | 22.29 | 52.81 | 81.03 | 112.43 | 727 | 806 | 897 | 805 | **762.9** |
| **TRT1** | **8** | **40** | **260** | 22.37 | 50.29 | 78.15 | 109.74 | 665 | 796 | 903 | 780 | **724.4** |
| **TRT1** | **8** | **40** | **607** | 22.78 | 52.39 | 79.93 | 111.47 | 705 | 787 | 901 | 792 | **742.2** |
| **TRT1** | **8** | **40** | **644** | 21.91 | 49.03 | 75.11 | 106.31 | 646 | 745 | 891 | 754 | **690.9** |
| **TRT1** | **9** | **44** | **215** | 21.10 | 52.52 | 81.74 | 115.75 | 748 | 835 | 972 | 845 | **787.5** |
| **TRT1** | **9** | **44** | **262** | 21.53 | 52.86 | 82.32 | 116.45 | 746 | 842 | 975 | 848 | **789.5** |
| **TRT1** | **9** | **44** | **268** | 21.85 | 55.46 | 86.68 | 119.12 | 800 | 892 | 927 | 868 | **841.9** |
| **TRT1** | **9** | **44** | **619** | 21.43 | 51.20 | 79.38 | 112.95 | 709 | 805 | 959 | 817 | **752.6** |
| **TRT1** | **10** | **48** | **295** | 20.58 | 53.01 | 83.47 | 114.95 | 772 | 870 | 899 | 843 | **816.8** |
| **TRT1** | **10** | **48** | **296** | 21.02 | 53.99 | 84.68 | 115.61 | 785 | 877 | 884 | 845 | **826.8** |
| **TRT1** | **10** | **48** | **663** | 20.73 | 52.06 | 82.45 | 111.51 | 746 | 868 | 830 | 811 | **801.6** |
| **TRT1** | **10** | **48** | **668** | 20.24 | 53.67 | 86.18 | 116.56 | 796 | 929 | 868 | 860 | **856.4** |
| **TRT2** | **1** | **3** | **231** | 29.45 | 60.46 | 91.18 | 124.35 | 738 | 878 | 948 | 847 | **801.7** |
| **TRT2** | **1** | **3** | **608** | 28.76 | 59.53 | 88.54 | 122.43 | 733 | 829 | 968 | 836 | **776.4** |
| **TRT2** | **1** | **3** | **624** | 29.27 | 58.39 | 87.03 | 119.62 | 693 | 818 | 931 | 807 | **750.1** |
| **TRT2** | **1** | **3** | **673** | 28.83 | 56.64 | 84.12 | 117.65 | 662 | 785 | 958 | 793 | **718.1** |
| **TRT2** | **2** | **7** | **238** | 28.12 | 60.31 | 90.58 | 124.56 | 766 | 865 | 971 | 861 | **811.2** |
| **TRT2** | **2** | **7** | **293** | 27.67 | 57.58 | 86.14 | 118.58 | 712 | 816 | 927 | 812 | **759.4** |
| **TRT2** | **2** | **7** | **605** | 27.86 | 57.28 | 85.39 | 118.88 | 700 | 803 | 957 | 813 | **747.1** |
| **TRT2** | **2** | **7** | **616** | 28.46 | 59.79 | 91.15 | 125.85 | 746 | 896 | 991 | 870 | **814.2** |
| **TRT2** | **3** | **11** | **226** | 26.82 | 55.23 | 83.14 | 116.17 | 676 | 797 | 944 | 798 | **731.4** |
| **TRT2** | **3** | **11** | **259** | 27.35 | 56.31 | 83.95 | 117.88 | 690 | 790 | 969 | 808 | **735.1** |
| **TRT2** | **3** | **11** | **632** | 26.91 | 54.55 | 81.38 | 112.33 | 658 | 767 | 884 | 763 | **707.4** |
| **TRT2** | **3** | **11** | **649** | 27.16 | 56.80 | 84.49 | 118.74 | 706 | 791 | 979 | 818 | **744.5** |
| **TRT2** | **4** | **20** | **219** | 26.03 | 55.05 | 82.19 | 111.86 | 691 | 775 | 848 | 766 | **729.4** |
| **TRT2** | **4** | **20** | **241** | 26.23 | 57.71 | 85.95 | 115.81 | 750 | 807 | 853 | 800 | **775.6** |
| **TRT2** | **4** | **20** | **273** | 26.52 | 58.89 | 88.95 | 118.87 | 771 | 859 | 855 | 825 | **810.8** |
| **TRT2** | **4** | **20** | **689** | 25.87 | 56.21 | 86.75 | 117.65 | 722 | 873 | 883 | 819 | **790.6** |
| **TRT2** | **5** | **24** | **284** | 25.69 | 58.98 | 90.85 | 123.13 | 793 | 911 | 922 | 870 | **846.2** |
| **TRT2** | **5** | **24** | **613** | 25.52 | 58.31 | 88.07 | 120.45 | 781 | 850 | 925 | 848 | **812.3** |
| **TRT2** | **5** | **24** | **657** | 25.24 | 55.43 | 84.68 | 115.21 | 719 | 836 | 872 | 803 | **771.9** |
| **TRT2** | **5** | **24** | **696** | 24.92 | 58.50 | 89.81 | 121.67 | 800 | 895 | 910 | 864 | **842.7** |
| **TRT2** | **6** | **28** | **216** | 24.21 | 53.42 | 82.92 | 114.47 | 695 | 843 | 901 | 806 | **762.5** |
| **TRT2** | **6** | **28** | **648** | 23.90 | 54.08 | 83.52 | 116.89 | 719 | 841 | 953 | 830 | **774.3** |
| **TRT2** | **6** | **28** | **660** | 24.72 | 55.11 | 84.23 | 118.68 | 724 | 832 | 984 | 839 | **772.9** |
| **TRT2** | **6** | **28** | **666** | 24.37 | 53.14 | 80.91 | 113.58 | 685 | 793 | 933 | 797 | **734.3** |
| **TRT2** | **7** | **32** | **214** | 23.46 | 53.51 | 84.02 | 116.34 | 715 | 872 | 923 | 829 | **786.5** |
| **TRT2** | **7** | **32** | **243** | 23.13 | 58.51 | 91.76 | 123.63 | 842 | 950 | 911 | 897 | **891.3** |
| **TRT2** | **7** | **32** | **604** | 23.68 | 54.57 | 84.24 | 115.85 | 735 | 848 | 903 | 823 | **786.5** |
| **TRT2** | **7** | **32** | **634** | 22.83 | 55.69 | 87.33 | 119.95 | 782 | 904 | 932 | 867 | **837.7** |
| **TRT2** | **8** | **36** | **217** | 22.02 | 53.47 | 84.18 | 116.82 | 749 | 877 | 933 | 846 | **807.3** |
| **TRT2** | **8** | **36** | **218** | 22.40 | 50.43 | 77.76 | 111.45 | 667 | 781 | 963 | 795 | **719.0** |
| **TRT2** | **8** | **36** | **253** | 22.25 | 51.23 | 81.19 | 114.14 | 690 | 856 | 941 | 820 | **765.5** |
| **TRT2** | **8** | **36** | **671** | 22.64 | 51.05 | 78.59 | 109.48 | 676 | 787 | 883 | 775 | **726.6** |
| **TRT2** | **9** | **45** | **247** | 21.14 | 49.11 | 75.96 | 107.99 | 666 | 767 | 915 | 775 | **711.9** |
| **TRT2** | **9** | **45** | **265** | 21.42 | 53.06 | 83.31 | 114.87 | 753 | 864 | 902 | 834 | **803.8** |
| **TRT2** | **9** | **45** | **294** | 21.84 | 52.39 | 81.12 | 114.29 | 727 | 821 | 948 | 825 | **769.9** |
| **TRT2** | **9** | **45** | **630** | 21.58 | 52.90 | 81.77 | 114.67 | 746 | 825 | 940 | 831 | **781.7** |
| **TRT2** | **10** | **49** | **270** | 21.01 | 48.91 | 77.12 | 111.83 | 664 | 806 | 992 | 811 | **728.7** |
| **TRT2** | **10** | **49** | **290** | 20.74 | 49.02 | 76.75 | 108.31 | 673 | 792 | 902 | 782 | **727.4** |
| **TRT2** | **10** | **49** | **652** | 20.31 | 51.26 | 80.88 | 114.98 | 737 | 846 | 974 | 845 | **786.6** |
| **TRT2** | **10** | **49** | **665** | 20.50 | 48.83 | 76.12 | 109.49 | 675 | 780 | 953 | 795 | **722.3** |
| **TRT3** | **1** | **4** | **233** | 28.76 | 56.06 | 82.65 | 113.12 | 650 | 760 | 871 | 753 | **699.9** |
| **TRT3** | **1** | **4** | **275** | 29.29 | 58.23 | 85.82 | 116.45 | 689 | 788 | 875 | 778 | **734.2** |
| **TRT3** | **1** | **4** | **606** | 29.36 | 58.30 | 86.12 | 117.75 | 689 | 795 | 904 | 789 | **737.1** |
| **TRT3** | **1** | **4** | **659** | 28.82 | 57.72 | 84.78 | 114.25 | 688 | 773 | 842 | 763 | **726.8** |
| **TRT3** | **2** | **8** | **224** | 28.38 | 59.53 | 88.77 | 123.42 | 742 | 835 | 990 | 849 | **784.3** |
| **TRT3** | **2** | **8** | **232** | 27.73 | 57.25 | 84.96 | 117.21 | 703 | 792 | 921 | 799 | **743.2** |
| **TRT3** | **2** | **8** | **235** | 28.18 | 56.86 | 86.14 | 119.33 | 683 | 837 | 948 | 814 | **752.7** |
| **TRT3** | **2** | **8** | **281** | 27.84 | 56.95 | 85.75 | 117.02 | 693 | 823 | 893 | 796 | **752.1** |
| **TRT3** | **3** | **12** | **202** | 26.88 | 57.17 | 85.42 | 118.45 | 721 | 807 | 944 | 818 | **760.3** |
| **TRT3** | **3** | **12** | **210** | 26.82 | 58.33 | 87.95 | 120.41 | 750 | 846 | 927 | 836 | **793.9** |
| **TRT3** | **3** | **12** | **234** | 27.35 | 56.78 | 85.22 | 117.65 | 701 | 813 | 927 | 806 | **751.6** |
| **TRT3** | **3** | **12** | **272** | 27.29 | 58.75 | 88.06 | 121.65 | 749 | 837 | 960 | 843 | **789.2** |
| **TRT3** | **4** | **16** | **245** | 26.02 | 56.26 | 84.95 | 118.16 | 720 | 820 | 949 | 823 | **765.3** |
| **TRT3** | **4** | **16** | **251** | 26.29 | 55.63 | 84.43 | 116.96 | 699 | 823 | 929 | 810 | **755.1** |
| **TRT3** | **4** | **16** | **602** | 26.38 | 54.52 | 82.92 | 114.77 | 670 | 811 | 910 | 789 | **734.3** |
| **TRT3** | **4** | **16** | **658** | 25.90 | 56.87 | 85.95 | 118.86 | 737 | 831 | 940 | 830 | **779.9** |
| **TRT3** | **5** | **25** | **249** | 25.54 | 54.23 | 82.05 | 114.87 | 683 | 795 | 938 | 798 | **733.9** |
| **TRT3** | **5** | **25** | **611** | 25.68 | 54.99 | 82.75 | 114.95 | 698 | 793 | 920 | 797 | **741.2** |
| **TRT3** | **5** | **25** | **672** | 24.99 | 53.62 | 81.65 | 113.15 | 682 | 801 | 900 | 787 | **735.8** |
| **TRT3** | **5** | **25** | **697** | 25.24 | 57.18 | 86.95 | 120.41 | 760 | 851 | 956 | 850 | **801.4** |
| **TRT3** | **6** | **29** | **236** | 24.09 | 52.73 | 81.31 | 115.91 | 682 | 817 | 989 | 820 | **743.1** |
| **TRT3** | **6** | **29** | **297** | 23.93 | 51.78 | 79.54 | 112.58 | 663 | 793 | 944 | 792 | **722.2** |
| **TRT3** | **6** | **29** | **669** | 24.69 | 53.80 | 81.96 | 118.87 | 693 | 805 | 1055 | 841 | **743.8** |
| **TRT3** | **6** | **29** | **699** | 24.37 | 52.85 | 80.62 | 115.96 | 678 | 793 | 1010 | 818 | **730.5** |
| **TRT3** | **7** | **33** | **229** | 22.87 | 54.08 | 83.96 | 116.96 | 743 | 854 | 943 | 840 | **793.4** |
| **TRT3** | **7** | **33** | **282** | 23.12 | 52.26 | 80.79 | 112.75 | 694 | 815 | 913 | 800 | **749.0** |
| **TRT3** | **7** | **33** | **636** | 23.61 | 54.84 | 84.15 | 117.76 | 744 | 837 | 960 | 841 | **786.2** |
| **TRT3** | **7** | **33** | **655** | 23.48 | 52.82 | 81.06 | 113.88 | 699 | 807 | 938 | 807 | **747.8** |
| **TRT3** | **8** | **37** | **252** | 22.59 | 54.38 | 84.39 | 117.89 | 757 | 857 | 957 | 851 | **802.6** |
| **TRT3** | **8** | **37** | **289** | 22.42 | 53.65 | 82.98 | 115.84 | 744 | 838 | 939 | 834 | **786.5** |
| **TRT3** | **8** | **37** | **621** | 22.08 | 51.49 | 79.79 | 112.75 | 700 | 809 | 942 | 810 | **749.5** |
| **TRT3** | **8** | **37** | **625** | 22.19 | 53.40 | 83.25 | 116.95 | 743 | 853 | 963 | 846 | **793.0** |
| **TRT3** | **9** | **41** | **221** | 21.15 | 53.55 | 82.91 | 112.81 | 771 | 839 | 854 | 818 | **802.1** |
| **TRT3** | **9** | **41** | **626** | 21.65 | 54.00 | 85.45 | 117.77 | 770 | 899 | 923 | 858 | **828.6** |
| **TRT3** | **9** | **41** | **675** | 21.35 | 53.41 | 83.31 | 114.64 | 763 | 854 | 895 | 833 | **804.7** |
| **TRT3** | **9** | **41** | **691** | 21.76 | 51.75 | 79.98 | 110.92 | 714 | 807 | 884 | 796 | **756.1** |
| **TRT3** | **10** | **50** | **209** | 20.96 | 51.57 | 79.95 | 111.96 | 729 | 811 | 915 | 813 | **766.1** |
| **TRT3** | **10** | **50** | **225** | 20.81 | 50.87 | 79.25 | 112.85 | 716 | 811 | 960 | 822 | **759.0** |
| **TRT3** | **10** | **50** | **261** | 20.32 | 51.96 | 81.12 | 113.51 | 753 | 833 | 925 | 832 | **789.6** |
| **TRT3** | **10** | **50** | **700** | 20.44 | 52.49 | 82.58 | 116.93 | 763 | 860 | 981 | 862 | **807.0** |

| **TRT** | **REP** | **ADG1** | **ADFI1** | **GF1** | **ADG2** | **ADFI2** | **GF2** | **ADG3** | **ADFI3** | **GF3** | **TADG** | **TADFI** | **TGF** |
| --- | --- | --- | --- | --- | --- | --- | --- | --- | --- | --- | --- | --- | --- |
|  |  |  |  |  |  |  |  |  |  |  |  |  |  |
| **Con** | **1** | 700 | 1675 | 0.418 | 818 | 2189 | 0.374 | 983 | 3081 | 0.319 | 825 | 2275 | 0.363 |
| **Con** | **2** | 623 | 1525 | 0.409 | 731 | 2051 | 0.357 | 963 | 3031 | 0.318 | 763 | 2160 | 0.353 |
| **Con** | **3** | 735 | 1688 | 0.435 | 841 | 2197 | 0.383 | 954 | 2911 | 0.328 | 836 | 2229 | 0.375 |
| **Con** | **4** | 716 | 1676 | 0.427 | 817 | 2033 | 0.402 | 938 | 3104 | 0.302 | 817 | 2234 | 0.366 |
| **Con** | **5** | 695 | 1675 | 0.415 | 798 | 2013 | 0.396 | 818 | 2765 | 0.296 | 765 | 2121 | 0.361 |
| **Con** | **6** | 690 | 1676 | 0.412 | 809 | 2244 | 0.361 | 906 | 2958 | 0.306 | 795 | 2254 | 0.353 |
| **Con** | **7** | 665 | 1630 | 0.408 | 784 | 2189 | 0.358 | 902 | 2903 | 0.311 | 776 | 2202 | 0.352 |
| **Con** | **8** | 711 | 1675 | 0.425 | 838 | 2184 | 0.384 | 856 | 2898 | 0.295 | 796 | 2216 | 0.359 |
| **Con** | **9** | 688 | 1675 | 0.411 | 806 | 2014 | 0.400 | 886 | 2925 | 0.303 | 787 | 2172 | 0.362 |
| **Con** | **10** | 670 | 1640 | 0.408 | 760 | 2179 | 0.349 | 928 | 2716 | 0.342 | 779 | 2145 | 0.363 |
| **TRT1** | **1** | 657 | 1586 | 0.414 | 743 | 2041 | 0.364 | 969 | 3113 | 0.311 | 781 | 2205 | 0.354 |
| **TRT1** | **2** | 642 | 1572 | 0.409 | 807 | 2013 | 0.401 | 917 | 2727 | 0.336 | 780 | 2071 | 0.376 |
| **TRT1** | **3** | 792 | 1824 | 0.434 | 862 | 2177 | 0.396 | 981 | 3070 | 0.320 | 873 | 2324 | 0.376 |
| **TRT1** | **4** | 746 | 1701 | 0.439 | 852 | 2248 | 0.379 | 925 | 3141 | 0.295 | 835 | 2322 | 0.360 |
| **TRT1** | **5** | 675 | 1651 | 0.409 | 775 | 2006 | 0.386 | 869 | 2541 | 0.342 | 767 | 2040 | 0.376 |
| **TRT1** | **6** | 714 | 1650 | 0.433 | 825 | 2091 | 0.395 | 839 | 2806 | 0.299 | 788 | 2149 | 0.367 |
| **TRT1** | **7** | 705 | 1650 | 0.427 | 799 | 2160 | 0.370 | 978 | 2874 | 0.340 | 820 | 2192 | 0.374 |
| **TRT1** | **8** | 686 | 1651 | 0.415 | 784 | 2191 | 0.358 | 898 | 3084 | 0.291 | 783 | 2268 | 0.345 |
| **TRT1** | **9** | 751 | 1731 | 0.434 | 843 | 2211 | 0.382 | 958 | 2925 | 0.328 | 845 | 2254 | 0.375 |
| **TRT1** | **10** | 775 | 1797 | 0.431 | 886 | 2180 | 0.406 | 870 | 2716 | 0.320 | 839 | 2204 | 0.381 |
| **TRT2** | **1** | 707 | 1692 | 0.418 | 828 | 2212 | 0.374 | 951 | 2926 | 0.325 | 821 | 2240 | 0.366 |
| **TRT2** | **2** | 731 | 1692 | 0.432 | 845 | 2155 | 0.392 | 962 | 3048 | 0.315 | 839 | 2260 | 0.371 |
| **TRT2** | **3** | 682 | 1626 | 0.420 | 786 | 2041 | 0.385 | 944 | 2755 | 0.343 | 797 | 2108 | 0.378 |
| **TRT2** | **4** | 733 | 1692 | 0.433 | 828 | 2191 | 0.378 | 860 | 2906 | 0.296 | 803 | 2227 | 0.360 |
| **TRT2** | **5** | 773 | 1727 | 0.448 | 873 | 2175 | 0.401 | 908 | 3068 | 0.296 | 846 | 2286 | 0.370 |
| **TRT2** | **6** | 706 | 1693 | 0.417 | 827 | 2209 | 0.374 | 943 | 2924 | 0.323 | 818 | 2239 | 0.365 |
| **TRT2** | **7** | 769 | 1726 | 0.445 | 893 | 2225 | 0.402 | 917 | 2939 | 0.312 | 854 | 2261 | 0.378 |
| **TRT2** | **8** | 696 | 1679 | 0.414 | 825 | 2216 | 0.372 | 930 | 3109 | 0.299 | 809 | 2294 | 0.353 |
| **TRT2** | **9** | 723 | 1692 | 0.427 | 819 | 2168 | 0.378 | 926 | 2882 | 0.321 | 817 | 2213 | 0.369 |
| **TRT2** | **10** | 687 | 1632 | 0.421 | 806 | 2223 | 0.363 | 955 | 2759 | 0.346 | 808 | 2169 | 0.373 |
| **TRT3** | **1** | 679 | 1640 | 0.414 | 779 | 2063 | 0.378 | 873 | 2956 | 0.295 | 771 | 2183 | 0.353 |
| **TRT3** | **2** | 705 | 1687 | 0.418 | 822 | 2022 | 0.406 | 938 | 2736 | 0.343 | 814 | 2119 | 0.384 |
| **TRT3** | **3** | 730 | 1687 | 0.433 | 826 | 2199 | 0.376 | 939 | 3092 | 0.304 | 825 | 2286 | 0.361 |
| **TRT3** | **4** | 706 | 1686 | 0.419 | 821 | 2042 | 0.402 | 932 | 2935 | 0.318 | 813 | 2188 | 0.372 |
| **TRT3** | **5** | 706 | 1686 | 0.419 | 810 | 2191 | 0.370 | 928 | 2905 | 0.320 | 808 | 2225 | 0.363 |
| **TRT3** | **6** | 679 | 1650 | 0.412 | 802 | 2228 | 0.360 | 999 | 2778 | 0.360 | 818 | 2183 | 0.374 |
| **TRT3** | **7** | 720 | 1685 | 0.427 | 828 | 2213 | 0.374 | 939 | 2928 | 0.321 | 822 | 2238 | 0.367 |
| **TRT3** | **8** | 736 | 1694 | 0.434 | 839 | 2182 | 0.385 | 950 | 2896 | 0.328 | 835 | 2222 | 0.376 |
| **TRT3** | **9** | 755 | 1705 | 0.443 | 850 | 2194 | 0.387 | 889 | 2909 | 0.306 | 826 | 2234 | 0.370 |
| **TRT3** | **10** | 740 | 1696 | 0.437 | 829 | 2187 | 0.379 | 945 | 2901 | 0.326 | 832 | 2226 | 0.374 |

Raw data for Table 2

| index | 11wC1F1 | 11wC1F5 | 11wC1F9 | 11wC1M1 | 11wC1M5 | 11wC1M9 |
| --- | --- | --- | --- | --- | --- | --- |
| Firmicutes | 34666.58 | 38607.08 | 33125.56 | 35779.5 | 36776.56 | 32718.47 |
| Bacteroidota | 10023.08 | 6302.953 | 9075.995 | 8726.398 | 7683.556 | 10486.01 |
| Proteobacteria | 244.4838 | 84.76044 | 361.4938 | 265.4703 | 393.6036 | 357.8949 |
| Spirochaetota | 88.83416 | 40.56022 | 1755.177 | 135.3378 | 89.07871 | 519.6509 |
| Actinobacteriota | 208.7983 | 189.281 | 429.1317 | 198.9582 | 213.3746 | 455.5737 |
| Planctomycetota | 0 | 0 | 96.05732 | 34.70198 | 22.26968 | 117.2145 |
| Verrucomicrobiota | 0 | 0 | 51.15478 | 0 | 3.107395 | 54.70009 |
| Fibrobacterota | 11.38898 | 8.840043 | 46.60769 | 9.832235 | 6.214789 | 31.25718 |
| Synergistota | 0 | 0 | 7.389028 | 0 | 0 | 7.032889 |
| WPS.2 | 0 | 0 | 0 | 0 | 0 | 1.562861 |
| Campilobacterota | 33.40774 | 5.720012 | 52.29157 | 35.85872 | 33.14556 | 91.42732 |
|  |  |  |  |  |  |  |
| index | 11wT1F1 | 11wT1F5 | 11wT1F9 | 11wT1M1 | 11wT1M5 | 11wT1M9 |
| Firmicutes | 33490.44 | 36424.36 | 33638.29 | 31421.37 | 32279.97 | 35719.96 |
| Bacteroidota | 10751.11 | 7798.682 | 10453.93 | 11564 | 11691.33 | 8518.551 |
| Proteobacteria | 242.8119 | 19.17201 | 245.7831 | 185.6119 | 485.8435 | 240.7158 |
| Spirochaetota | 151.7575 | 145.9547 | 160.0777 | 1305.512 | 149.6791 | 48.03984 |
| Actinobacteriota | 371.5023 | 553.5147 | 362.654 | 436.6241 | 263.3697 | 532.0543 |
| Planctomycetota | 148.1153 | 71.12198 | 83.58043 | 38.61725 | 41.71383 | 0 |
| Verrucomicrobiota | 12.74763 | 6.184516 | 9.208001 | 19.93147 | 5.725421 | 0 |
| Fibrobacterota | 9.712461 | 11.13216 | 24.08251 | 14.9486 | 13.08668 | 3.099345 |
| Synergistota | 0 | 0 | 3.541545 | 1.868578 | 0 | 0 |
| WPS.2 | 0 | 0 | 0 | 0 | 0 | 0 |
| Campilobacterota | 29.13743 | 17.31667 | 66.58103 | 24.29152 | 55.61847 | 1.033115 |
|  |  |  |  |  |  |  |
| index | 11wT2F1 | 11wT2F5 | 11wT2F9 | 11wT2M1 | 11wT2M5 | 11wT2M9 |
| Firmicutes | 34018.62 | 35304.83 | 33111.28 | 3599.8 | 36596.62 | 37177.01 |
| Bacteroidota | 9933.019 | 9056.762 | 10945.59 | 858.0759 | 7933.73 | 7275.401 |
| Proteobacteria | 44.84658 | 62.46699 | 92.24588 | 36.96928 | 204.4837 | 272.2304 |
| Spirochaetota | 74.24602 | 156.8828 | 46.67865 | 14.75528 | 190.1758 | 220.4318 |
| Actinobacteriota | 834.6447 | 506.4119 | 609.6009 | 11.9988 | 178.8487 | 186.475 |
| Planctomycetota | 24.91475 | 15.25911 | 7.224072 | 5.59401 | 68.55869 | 68.48928 |
| Verrucomicrobiota | 2.491475 | 4.768457 | 0 | 0 | 0 | 0 |
| Fibrobacterota | 9.965918 | 30.04139 | 13.33677 | 1.540384 | 16.69253 | 17.84172 |
| Synergistota | 0 | 0 | 0 | 0 | 0 | 0 |
| WPS.2 | 39.86363 | 2.861083 | 21.67222 | 0.891804 | 14.30792 | 14.96406 |
| Campilobacterota | 0 | 10.01378 | 0 | 0 | 6.557793 | 3.453239 |
|  |  |  |  |  |  |  |
| index | 16WC1 | 16WC10 | 16WC2 | 16WC5 | 16WC6 | 16WC9 |
| Firmicutes | 31664.12 | 36021.35 | 36779.57 | 31500.28 | 32285.59 | 32532.77 |
| Bacteroidota | 9055.66 | 7231.302 | 6792.43 | 9211.101 | 8842.649 | 8632.201 |
| Proteobacteria | 2151.093 | 532.1193 | 636.2716 | 2311.093 | 2036.531 | 2066.053 |
| Spirochaetota | 1919.641 | 1109.698 | 592.624 | 1765.48 | 1652.288 | 1574.431 |
| Actinobacteriota | 94.57176 | 141.655 | 406.9679 | 79.29134 | 96.86783 | 98.83503 |
| Planctomycetota | 26.54645 | 74.27752 | 27.04923 | 23.28837 | 20.29611 | 25.89403 |
| Verrucomicrobiota | 19.90984 | 55.20078 | 9.836098 | 24.95179 | 27.21523 | 19.69406 |
| Fibrobacterota | 9.125364 | 60.8832 | 33.81152 | 7.762805 | 11.07061 | 11.30584 |
| Synergistota | 0 | 13.39432 | 3.688532 | 2.772423 | 2.76765 | 4.376457 |
| WPS.2 | 0 | 0 | 0 | 3.326911 | 0 | 0.729412 |
| Campilobacterota | 271.2716 | 70.21866 | 38.11484 | 305.5211 | 250.9338 | 255.2935 |
|  |  |  |  |  |  |  |
| index | 16wTRT1-1 | 16wTRT1-10 | 16wTRT1-2 | 16wTRT1-5 | 16wTRT1-6 | 16wTRT1-9 |
| Firmicutes | 34536.68 | 34379.04 | 34648.97 | 31692.29 | 36655.28 | 35061.42 |
| Bacteroidota | 8113.823 | 8186.188 | 7991.884 | 10373.51 | 6033.448 | 7649.714 |
| Proteobacteria | 444.848 | 363.5447 | 319.1711 | 181.5479 | 179.4317 | 283.7912 |
| Spirochaetota | 1207.511 | 1326.009 | 1755.773 | 1975.425 | 1806.784 | 1321.609 |
| Actinobacteriota | 545.3576 | 620.8372 | 75.64553 | 152.5922 | 115.3172 | 576.9322 |
| Planctomycetota | 161.4668 | 148.6579 | 193.7587 | 327.7055 | 256.4582 | 145.7455 |
| Verrucomicrobiota | 83.2927 | 78.61715 | 78.96334 | 100.196 | 97.50757 | 66.54794 |
| Fibrobacterota | 18.14755 | 41.92915 | 22.56095 | 90.54413 | 54.31927 | 32.99897 |
| Synergistota | 31.17658 | 22.87046 | 12.60758 | 23.44037 | 10.24051 | 29.69907 |
| WPS.2 | 1.861287 | 1.905869 | 35.83209 | 0 | 32.05728 | 0 |
| Campilobacterota | 107.4894 | 112.4464 | 114.7954 | 313.917 | 113.9815 | 109.4466 |
|  |  |  |  |  |  |  |
| index | 16wTRT2-1 | 16wTRT2-10 | 16wTRT2-2 | 16wTRT2-5 | 16wTRT2-6 | 16wTRT2-9 |
| Firmicutes | 31490.53 | 30246.21 | 37848.63 | 36782.64 | 34605 | 33822.6 |
| Bacteroidota | 8178.55 | 7680.597 | 5948.086 | 6785.347 | 4905 | 7389.883 |
| Proteobacteria | 5033.537 | 6520.993 | 623.5646 | 757.5748 | 5264 | 3509.752 |
| Spirochaetota | 271.8329 | 447.1779 | 368.3006 | 428.7966 | 339 | 212.2972 |
| Actinobacteriota | 235.6446 | 191.4217 | 273.2753 | 233.3761 | 173 | 265.9422 |
| Planctomycetota | 0 | 0 | 18.01132 | 37.44278 | 0 | 0 |
| Verrucomicrobiota | 5.049505 | 12.65599 | 21.11671 | 15.38743 | 0 | 9.701733 |
| Fibrobacterota | 8.415857 | 13.71064 | 3.726483 | 17.43908 | 7.999989 | 0 |
| Synergistota | 0 | 0 | 0 | 2.564571 | 0 | 0 |
| WPS.2 | 10.94065 | 25.31193 | 0 | 5.642056 | 10.00001 | 23.96906 |
| Campilobacterota | 60.59433 | 126.5598 | 27.9486 | 135.9224 | 29.00002 | 53.0743 |
|  |  |  |  |  |  |  |
|  |  |  |  |  |  |  |
| index | 16wTRT3-1 | 16wTRT3-10 | 16wTRT3-2 | 16wTRT3-5 | 16wTRT3-6 | 16wTRT3-9 |
| Firmicutes | 34326.74 | 33626.29 | 32497.4 | 33231.92 | 32364.52 | 35802.09 |
| Bacteroidota | 7886.245 | 6689.102 | 6954.925 | 6463.825 | 7863.284 | 6608.548 |
| Proteobacteria | 1526.986 | 3629.563 | 4556.818 | 4208.367 | 3255.296 | 1020.362 |
| Spirochaetota | 793.9373 | 598.1971 | 642.3395 | 562.8336 | 773.6038 | 1252.613 |
| Actinobacteriota | 417.2648 | 447.9435 | 274.3572 | 335.5354 | 476.1574 | 135.1047 |
| Planctomycetota | 22.68391 | 14.55583 | 16.5918 | 11.33916 | 25.9354 | 0 |
| Verrucomicrobiota | 100.8838 | 46.01514 | 69.92256 | 55.66487 | 116.304 | 20.58739 |
| Fibrobacterota | 55.51592 | 37.56342 | 38.51666 | 34.53285 | 76.99568 | 6.433564 |
| Synergistota | 0 | 3.286797 | 0 | 2.577076 | 2.431442 | 19.30065 |
| WPS.2 | 88.34793 | 71.37044 | 73.47796 | 75.25066 | 113.062 | 0 |
| Campilobacterota | 63.87314 | 153.0708 | 204.4346 | 360.2754 | 208.2935 | 74.62925 |

Raw data for Table 3.

| index | 11wC1F1 | 11wC1F5 | 11wC1F9 | 11wC1M1 | 11wC1M5 | 11wC1M9 |
| --- | --- | --- | --- | --- | --- | --- |
| g_Eubacterium_siraeum | 210.3168 | 180.4409 | 131.2973 | 57.2583 | 43.50357 | 358.6764 |
| o_Bacteroidales | 37.20405 | 59.80033 | 7.957398 | 54.36645 | 75.09543 | 14.84715 |
| g_Megasphaera | 1244.438 | 1772.169 | 417.1956 | 857.1395 | 957.0782 | 663.434 |
| g_Acidaminococcus | 50.87083 | 15.60007 | 34.1032 | 38.75057 | 50.23623 | 68.76582 |
| g_Streptococcus | 31.88918 | 18.7201 | 15.91484 | 25.44812 | 21.23389 | 48.44867 |
| g_Prevotella | 6633.71 | 3646.259 | 1413.577 | 4581.821 | 3809.668 | 2950.68 |
| f_Atopobiaceae | 17.46313 | 28.08014 | 48.31285 | 28.91833 | 27.44868 | 47.66725 |
| g_Prevotellaceae_UCG.003 | 98.70462 | 66.04034 | 131.2973 | 134.1811 | 125.3317 | 64.07728 |
| g_Intestinibacter | 450.245 | 326.5617 | 247.2481 | 398.4947 | 433.4818 | 265.6862 |
| g_Colidextribacter | 87.31565 | 52.00029 | 79.0057 | 133.6027 | 127.4033 | 157.8489 |
| g_Terrisporobacter | 1095.621 | 674.9636 | 2882.856 | 2397.33 | 2646.984 | 2586.533 |
| g_Peptococcus | 96.42683 | 88.40047 | 81.84764 | 139.9648 | 149.673 | 115.6517 |
| g_Romboutsia | 305.2251 | 145.6008 | 1210.095 | 819.5457 | 881.9828 | 931.4645 |
| g_WPS.2 | 0 | 0 | 0 | 0 | 0 | 1.562861 |
| g_Clostridium_sensu_stricto_6 | 96.42683 | 173.1609 | 155.7379 | 41.64242 | 56.45102 | 30.47576 |
| c_Bacilli_._._. | 2721.97 | 4048.221 | 266.0048 | 5820.105 | 6397.612 | 121.9031 |
| g_Lactobacillus | 5784.091 | 14521.6 | 518.3684 | 6589.332 | 7062.595 | 193.7946 |
|  |  |  |  |  |  |  |
| index | 11wT1F1 | 11wT1F5 | 11wT1F9 | 11wT1M1 | 11wT1M5 | 11wT1M9 |
| g_Eubacterium_siraeum | 53.41863 | 110.7029 | 47.4567 | 72.8745 | 94.87854 | 48.03984 |
| o_Bacteroidales | 15.78279 | 121.2166 | 83.58043 | 49.82873 | 65.43347 | 17.0464 |
| g_Megasphaera | 1390.705 | 2382.278 | 894.5939 | 977.8886 | 906.2537 | 1399.354 |
| g_Acidaminococcus | 106.8373 | 108.2291 | 148.0365 | 63.53159 | 140.682 | 352.2923 |
| g_Streptococcus | 30.95854 | 59.98987 | 212.4926 | 85.33167 | 218.3842 | 53.72199 |
| g_Prevotella | 5907.614 | 5194.998 | 5811.673 | 6488.322 | 7514.217 | 5063.814 |
| f_Atopobiaceae | 60.09596 | 42.05474 | 58.08131 | 57.9259 | 32.71674 | 39.25839 |
| g_Prevotellaceae_UCG.003 | 98.33885 | 135.441 | 325.1137 | 335.0981 | 214.2946 | 127.0732 |
| g_Intestinibacter | 493.5153 | 466.9314 | 384.6116 | 357.521 | 261.7339 | 430.2924 |
| g_Colidextribacter | 225.8151 | 103.2815 | 100.5798 | 195.5777 | 91.60687 | 74.90084 |
| g_Terrisporobacter | 2814.19 | 3426.225 | 2976.313 | 3046.403 | 2576.443 | 2803.874 |
| g_Peptococcus | 239.7768 | 142.8625 | 86.41364 | 209.9035 | 94.87854 | 173.5633 |
| g_Romboutsia | 869.8737 | 876.3468 | 1508.697 | 828.4024 | 1154.083 | 1077.023 |
| g_WPS.2 | 0 | 0 | 0 | 0 | 0 | 0 |
| g_Clostridium_sensu_stricto_6 | 38.84989 | 58.13449 | 21.24926 | 18.68578 | 27.80923 | 12.91395 |
| c_Bacilli_._._. | 66.77326 | 341.3856 | 795.4306 | 69.13733 | 382.7858 | 203.0071 |
| g_Lactobacillus | 284.697 | 1655.597 | 2513.787 | 192.4634 | 1155.719 | 499.5112 |
|  |  |  |  |  |  |  |
| index | 11wT2F1 | 11wT2F5 | 11wT2F9 | 11wT2M1 | 11wT2M5 | 11wT2M9 |
| g_Eubacterium_siraeum | 39.86363 | 87.26305 | 73.90788 | 76.20694 | 73.32796 | 59.85614 |
| o_Bacteroidales | 133.5432 | 61.5133 | 143.9258 | 142.6853 | 149.0406 | 139.8562 |
| g_Megasphaera | 1643.378 | 1072.907 | 1226.426 | 3700.09 | 4266.138 | 3864.751 |
| g_Acidaminococcus | 170.417 | 32.42564 | 130.0334 | 89.1783 | 84.05889 | 71.94249 |
| g_Streptococcus | 2.491475 | 92.03156 | 15.00383 | 31.61777 | 38.15438 | 35.68349 |
| g_Prevotella | 3671.938 | 4953.491 | 5193 | 5327.188 | 4338.274 | 3823.888 |
| f_Atopobiaceae | 45.34486 | 40.05517 | 30.5634 | 15.40352 | 22.65418 | 23.59715 |
| g_Prevotellaceae_UCG.003 | 126.567 | 142.1005 | 111.6953 | 50.26414 | 54.84696 | 60.4317 |
| g_Intestinibacter | 241.175 | 315.1961 | 117.2523 | 192.1387 | 206.8683 | 219.8563 |
| g_Colidextribacter | 100.6556 | 53.4069 | 92.80161 | 29.99635 | 42.32751 | 47.19429 |
| g_Terrisporobacter | 2303.619 | 3117.628 | 1166.966 | 517.2343 | 619.4127 | 526.6191 |
| g_Peptococcus | 128.5602 | 43.39312 | 88.35602 | 48.64271 | 63.19322 | 50.072 |
| g_Romboutsia | 904.9044 | 673.3085 | 531.8032 | 119.9854 | 162.7524 | 131.7987 |
| g_WPS.2 | 39.86363 | 2.861083 | 21.67222 | 8.917816 | 14.30792 | 14.96406 |
| g_Clostridium_sensu_stricto_6 | 14.45055 | 347.1449 | 7.77976 | 424.8131 | 440.564 | 444.8924 |
| c_Bacilli_._._. | 953.239 | 350.4828 | 1921.604 | 3186.098 | 3343.279 | 3248.923 |
| g_Lactobacillus | 6263.074 | 1151.586 | 3683.723 | 10727.34 | 10941.97 | 11376.12 |

Raw data for Table 4.

| X.OTU.ID |  | 11wT2F1 | 11wT2F5 | 11wT2F9 | 11wT2M1 | 11wT2M5 | 11wT2M9 |
| --- | --- | --- | --- | --- | --- | --- | --- |
| PWY.5088 | amonia, CO2 생성. L-glutamate degradation VIII (to propanoate) | 206.1219 | 1569.014 | 78.66284 | 1325.794 | 435.9174 | 1452.112 |
| PWY.7332 | LPS synthesis, superpathway of UDP-N-acetylglucosamine-derived O-antigen building blocks biosynthesis | 3846.986 | 5924.465 | 3272.256 | 2400.213 | 3551.709 | 3569.763 |
| PWY.7198 | pyrimidine deoxyribonucleotides de novo biosynthesis IV | 4058.784 | 7131.975 | 4874.707 | 1355.646 | 2950.929 | 2678.885 |
| PWY.7210 | pyrimidine deoxyribonucleotides biosynthesis from CTP | 5429.324 | 9269.02 | 6378.228 | 1891.149 | 4056.801 | 3702.027 |
| PWY.5705 | purin product, allantoin degradation to glyoxylate III | 568.3487 | 949.674 | 493.7271 | 261.3061 | 423.7495 | 398.9129 |
|  |  |  |  |  |  |  |  |
| X.OTU.ID |  | 16WC1 | 16WC10 | 16WC2 | 16WC5 | 16WC6 | 16WC9 |
| PWY.5088 | amonia, CO2 생성. L-glutamate degradation VIII (to propanoate) | 2042.823 | 2559.508 | 1189.718 | 3705.807 | 4615.369 | 4526.385 |
| PWY.7332 | LPS synthesis, superpathway of UDP-N-acetylglucosamine-derived O-antigen building blocks biosynthesis | 10960.46 | 16241.65 | 6049.538 | 16691.15 | 19719.83 | 23233.44 |
| PWY.7198 | pyrimidine deoxyribonucleotides de novo biosynthesis IV | 1507.627 | 1847.264 | 1969.998 | 2022.549 | 2773.77 | 3620.704 |
| PWY.7210 | pyrimidine deoxyribonucleotides biosynthesis from CTP | 2107.989 | 2608.964 | 2761.178 | 2835.287 | 3878.183 | 5057.525 |
| PWY.5705 | purin product, allantoin degradation to glyoxylate III | 277.0027 | 825.9165 | 452.9316 | 422.4969 | 554.9168 | 701.8729 |
|  |  |  |  |  |  |  |  |
| X.OTU.ID |  | 16wTRT1-1 | 16wTRT1-10 | 16wTRT1-2 | 16wTRT1-5 | 16wTRT1-6 | 16wTRT1-9 |
| PWY.5088 | amonia, CO2 생성. L-glutamate degradation VIII (to propanoate) | 1372.901 | 1402.697 | 644.8545 | 901.7158 | 57.44796 | 1078.903 |
| PWY.7332 | LPS synthesis, superpathway of UDP-N-acetylglucosamine-derived O-antigen building blocks biosynthesis | 6893.442 | 7233.073 | 17346.68 | 17299.13 | 7164.31 | 5746.881 |
| PWY.7198 | pyrimidine deoxyribonucleotides de novo biosynthesis IV | 918.8104 | 1012.086 | 860.083 | 1504.601 | 1043.087 | 806.5903 |
| PWY.7210 | pyrimidine deoxyribonucleotides biosynthesis from CTP | 1302.076 | 1432.585 | 1217.235 | 2120.749 | 1477.495 | 1142.462 |
| PWY.5705 | purin product, allantoin degradation to glyoxylate III | 193.0899 | 201.8265 | 211.7747 | 399.0691 | 269.2927 | 192.1163 |
|  |  |  |  |  |  |  |  |
| X.OTU.ID |  | 16wTRT2-1 | 16wTRT2-10 | 16wTRT2-2 | 16wTRT2-5 | 16wTRT2-6 | 16wTRT2-9 |
| PWY.5088 | amonia, CO2 생성. L-glutamate degradation VIII (to propanoate) | 497.8782 | 1056.982 | 1418.309 | 1902.458 | 809.8922 | 604.9952 |
| PWY.7332 | LPS synthesis, superpathway of UDP-N-acetylglucosamine-derived O-antigen building blocks biosynthesis | 901.3737 | 1846.759 | 5774.152 | 6603.631 | 1042.031 | 1847.395 |
| PWY.7198 | pyrimidine deoxyribonucleotides de novo biosynthesis IV | 418.8852 | 685.6664 | 1591.727 | 1237.588 | 712.8713 | 382.4005 |
| PWY.7210 | pyrimidine deoxyribonucleotides biosynthesis from CTP | 594.4897 | 973.4414 | 2226.569 | 1745.305 | 1005.551 | 544.0127 |
| PWY.5705 | purin product, allantoin degradation to glyoxylate III | 60.66408 | 113.7968 | 501.3725 | 451.8128 | 93.27335 | 104.8743 |
|  |  |  |  |  |  |  |  |
| X.OTU.ID |  | 16wTRT3-1 | 16wTRT3-10 | 16wTRT3-2 | 16wTRT3-5 | 16wTRT3-6 | 16wTRT3-9 |
| PWY.5088 | amonia, CO2 생성. L-glutamate degradation VIII (to propanoate) | 945.7785 | 1217.246 | 789.6238 | 983.3768 | 1437.508 | 1551.328 |
| PWY.7332 | LPS synthesis, superpathway of UDP-N-acetylglucosamine-derived O-antigen building blocks biosynthesis | 3965.178 | 3225.16 | 2483.004 | 2484.893 | 5089.242 | 3794.143 |
| PWY.7198 | pyrimidine deoxyribonucleotides de novo biosynthesis IV | 260.9696 | 513.6658 | 364.8829 | 434.3517 | 441.7816 | 1515.095 |
| PWY.7210 | pyrimidine deoxyribonucleotides biosynthesis from CTP | 371.2808 | 729.595 | 518.789 | 617.6019 | 628.5287 | 2127.714 |
| PWY.5705 | purin product, allantoin degradation to glyoxylate III | 110.9406 | 205.568 | 119.7218 | 161.7576 | 200.3323 | 341.7076 |

Raw data for Figure 3A

|  |  | T1 증가 | T1 감소 | T2 증가 | T2 감소 | 공통증가 | 공통감소 |
| --- | --- | --- | --- | --- | --- | --- | --- |
| 9101 | Carbohydrate metabolism | 7 | 21 | 10 | 9 | 2 | 0 |
| 9102 | Energy metabolism | 0 | 0 | 2 | 14 | 0 | 0 |
| 9103 | Lipid metabolism | 2 | 2 | 5 | 0 | 0 | 0 |
| 9104 | Nucleotide metabolism | 0 | 5 | 0 | 0 | 0 | 0 |
| 9105 | Amino acid metabolism | 4 | 11 | 7 |  | 1 | 0 |
| 9107 | Glycan biosynthesis and metabolism | 4 | 8 | 18 | 1 | 0 | 0 |
| 9108 | Metabolism of cofactors and vitamins | 2 | 12 | 13 | 1 | 2 | 0 |
| 9131 | Membrane transport | 2 | 8 | 11 | 1 | 1 | 0 |
| 9132 | Signal transduction | 5 | 9 | 3 | 0 | 0 | 0 |
| 9181 | Protein families: metabolism | 9 | 51 | 29 | 19 | 1 | 0 |
|  | no included in the chart due to poorly defined functions | 10 | 36 | 23 | 51 | 3 | 2 |
|  | Sum | 45 | 163 | 121 | 96 | 10 | 2 |

Raw data for Figure 3B

| X.OTU.ID | log(T1/c,2) | log(T2/c,2) | Log(T2/T1) |
| --- | --- | --- | --- |
| PWY.6467 | 0.164111 | 0.402341 | 0.23823 |
| PWY.1269 | 0.15966 | 0.383413 | 0.223753 |
| NAGLIPASYN.PWY | 0.137464 | 0.368384 | 0.23092 |
| PWY.7234 | -1.65689 | -0.15922 | 1.497666 |
| PWY.5484 | -0.5651 | 0.023184 | 0.588281 |
| PWY.7392 | -0.73374 | -0.03319 | 0.700544 |
| PWY.2941 | -1.20489 | 0.159083 | 1.363972 |
| ANAEROFRUCAT.PWY | -0.43585 | 0.042958 | 0.478807 |
| GLYCOLYSIS | -0.33845 | 0.069025 | 0.407477 |
| P23.PWY | -0.48452 | 0.013634 | 0.498152 |
| PENTOSE.P.PWY | -1.11862 | -0.04521 | 1.073408 |
| P122.PWY | -1.39607 | -0.12676 | 1.269302 |
| P461.PWY | -0.61242 | -0.01774 | 0.594677 |
| PWY.5910 | -2.36648 | -0.19806 | 2.168421 |
| P124.PWY | -1.0747 | -0.0772 | 0.997504 |
| PWY.6628 | 0.306967 | 0.31783 | 0.010863 |
| PWY.5154 | 0.3558 | 0.138457 | -0.21734 |
| FASYN.ELONG.PWY | -0.41522 | -0.08211 | 0.333109 |
| GLYCOLYSIS.E.D | -0.87722 | -0.07194 | 0.805287 |
| PWY.6630 | 0.308308 | 0.31485 | 0.006542 |
| PWY.6892 | 0.288771 | 0.285456 | -0.00331 |
| PWY.6901 | -0.25787 | -0.48653 | -0.22866 |

Raw data for Figure 3C

|  |  | T1 Increase | T1 Decrease | T2 Increase | T2 decrease | T1&T2 increase | T1&T2 decrease |
| --- | --- | --- | --- | --- | --- | --- | --- |
| 9101 | Carbohydrate metabolism | 40 | 11 | 19 | 24 | 16 | 6 |
| 9102 | Energy metabolism | 20 | 4 | 8 | 1 | 7 | 1 |
| 9103 | Lipid metabolism | 9 | 3 | 4 | 2 | 2 | 1 |
| 9104 | Nucleotide metabolism | 3 | 0 | 4 | 5 | 1 | 0 |
| 9105 | Amino acid metabolism | 18 | 7 | 12 | 8 | 9 | 3 |
| 9106 | Metabolism of other amino acids | 4 | 0 | 1 | 2 | 0 | 0 |
| 9107 | Glycan biosynthesis and metabolism | 8 | 3 | 5 | 6 | 2 | 1 |
| 9108 | Metabolism of cofactors and vitamins | 12 | 2 | 13 | 5 | 7 | 1 |
| 9109 | Metabolism of terpenoids and polyketides | 3 | 1 | 1 | 0 | 0 | 0 |
| 9111 | Xenobiotics biodegradation and metabolism | 10 | 0 | 5 | 5 | 5 | 6 |
| 9121 | Transcription | 1 | 0 | 1 | 0 | 0 | 0 |
| 9122 | Translation | 0 | 0 | 1 | 1 | 0 | 0 |
| 9123 | Folding, sorting and degradation | 3 | 2 | 7 | 2 | 3 | 1 |
| 9124 | Replication and repair | 3 | 0 | 6 | 1 | 0 | 0 |
| 9131 | Membrane transport | 21 | 11 | 17 | 22 | 11 | 8 |
| 9132 | Signal transduction | 30 | 7 | 22 | 9 | 20 | 5 |
| 9140 | Cellular Processes | 5 | 2 | 6 | 4 | 5 | 1 |
| 9181 | Protein families: metabolism | 10 | 6 | 5 | 6 | 2 | 4 |
| 9182 | Protein families: genetic information processing | 46 | 10 | 53 | 9 | 31 | 3 |
| 9183 | Protein families: signaling and cellular processes | 34 | 27 | 39 | 18 | 22 | 8 |
|  | not included in the chart due to poorly defined function | 69 | 13 | 79 | 27 | 46 | 0 |
|  | sum | 349 | 109 | 308 | 157 | 189 | 47 |

Raw data for Figure 3D

| X.OTU.ID | log(T1/C) | log(T2/c) | Log(T3/c) | Log (T2/T1) | Log (T3/T1) | Log (T2/T3) |
| --- | --- | --- | --- | --- | --- | --- |
| PWY.3781 | 0.724 | -1.641 | -0.564 | -2.365 | -1.288 | -1.077 |
| PWY.6901 | 0.173 | -1.625 | -0.740 | -1.798 | -0.913 | -0.885 |
| REDCITCYC | 0.651 | -1.391 | -0.358 | -2.041 | -1.009 | -1.033 |
| PWY.6588 | 0.442 | -0.774 | -0.166 | -1.216 | -0.608 | -0.608 |
| SULFATE.CYS.PWY | 0.108 | -1.155 | -0.508 | -1.263 | -0.616 | -0.647 |
| PWY0.1241 | -1.293 | 0.569 | 0.431 | 1.862 | 1.724 | 0.138 |
| PWY0.845 | -0.199 | -1.450 | -0.405 | -1.251 | -0.206 | -1.045 |
| GLYCOLYSIS.E.D | -0.229 | -1.013 | -0.778 | -0.785 | -0.549 | -0.235 |
| PWY.5971 | -1.439 | -0.342 | 0.005 | 1.096 | 1.444 | -0.348 |
| PWY.6282 | -1.627 | -0.309 | 0.023 | 1.317 | 1.650 | -0.332 |
| FASYN.INITIAL.PWY | -1.660 | -0.311 | 0.025 | 1.349 | 1.685 | -0.336 |
| PWY.7664 | -1.562 | -0.315 | 0.021 | 1.247 | 1.583 | -0.336 |
| GLUCUROCAT.PWY | 0.623 | -0.323 | 0.205 | -0.945 | -0.417 | -0.528 |
| PWYG.321 | -1.537 | -0.323 | 0.035 | 1.214 | 1.572 | -0.358 |
| PWY.5989 | -1.600 | -0.303 | 0.020 | 1.297 | 1.620 | -0.323 |
| TEICHOICACID.PWY | 0.143 | -1.477 | -1.268 | -1.620 | -1.411 | -0.210 |
| PWY0.862 | -1.527 | -0.308 | 0.022 | 1.219 | 1.550 | -0.331 |
| PWY.7254 | -0.861 | 0.230 | 0.160 | 1.090 | 1.021 | 0.069 |
| P441.PWY | 0.180 | -0.823 | -0.406 | -1.003 | -0.585 | -0.417 |
| PWY.2941 | -0.342 | -0.899 | -0.688 | -0.557 | -0.347 | -0.210 |
| SALVADEHYPOX.PWY | 0.474 | -0.700 | -0.210 | -1.174 | -0.684 | -0.490 |
| GALACTUROCAT.PWY | 0.500 | -0.400 | 0.014 | -0.900 | -0.486 | -0.414 |
| P124.PWY | 0.009 | -0.971 | -0.551 | -0.980 | -0.560 | -0.420 |
| PWY.5910 | -0.090 | -1.302 | -0.988 | -1.212 | -0.898 | -0.314 |
| PWY.6507 | 0.168 | -0.828 | -0.492 | -0.995 | -0.659 | -0.336 |
| PWY.6471 | -0.026 | -0.916 | -0.535 | -0.890 | -0.508 | -0.382 |
| PWY.4984 | 0.295 | -0.988 | -1.129 | -1.284 | -1.424 | 0.140 |
| PWY.922 | -0.092 | -1.386 | -1.056 | -1.294 | -0.964 | -0.331 |
| PWY.6263 | -0.984 | 0.101 | 0.015 | 1.085 | 0.999 | 0.086 |
| P122.PWY | -0.057 | -1.020 | -0.573 | -0.963 | -0.516 | -0.447 |
| PWY.7220 | 0.079 | -0.648 | -0.368 | -0.727 | -0.447 | -0.280 |
| PWY.7222 | 0.079 | -0.648 | -0.368 | -0.727 | -0.447 | -0.280 |
| PWY.6353 | 0.398 | -0.586 | -0.206 | -0.984 | -0.605 | -0.379 |
| PWY.7392 | 0.013 | -0.717 | -0.403 | -0.731 | -0.416 | -0.315 |
| KDO.NAGLIPASYN.PWY | -2.033 | 0.482 | 0.595 | 2.515 | 2.627 | -0.112 |
| GALACT.GLUCUROCAT.PWY | 0.529 | -0.317 | 0.156 | -0.846 | -0.373 | -0.473 |
| HOMOSER.METSYN.PWY | -0.600 | 0.260 | 0.470 | 0.860 | 1.070 | -0.210 |
| HSERMETANA.PWY | -0.578 | 0.153 | 0.443 | 0.731 | 1.020 | -0.290 |
| PWY.5840 | -1.779 | 0.013 | 0.244 | 1.792 | 2.022 | -0.230 |
| PWY.5897 | -1.833 | 0.047 | 0.273 | 1.879 | 2.106 | -0.226 |
| PWY.5898 | -1.833 | 0.047 | 0.273 | 1.879 | 2.106 | -0.226 |
| PWY.5899 | -1.833 | 0.047 | 0.273 | 1.879 | 2.106 | -0.226 |
| PWY.5838 | -1.756 | 0.008 | 0.240 | 1.764 | 1.996 | -0.232 |
| GLCMANNANAUT.PWY | 0.326 | -0.716 | -0.321 | -1.042 | -0.647 | -0.395 |
| PPGPPMET.PWY | -2.633 | 0.627 | 0.809 | 3.260 | 3.442 | -0.182 |
| P161.PWY | 0.085 | -0.670 | -0.291 | -0.755 | -0.376 | -0.379 |
| PWY.5861 | -1.926 | 0.136 | 0.341 | 2.062 | 2.267 | -0.205 |
| MET.SAM.PWY | -0.567 | 0.160 | 0.382 | 0.727 | 0.949 | -0.223 |
| PWY.7234 | -0.287 | -0.736 | -0.407 | -0.449 | -0.120 | -0.329 |
| P461.PWY | -0.338 | -0.578 | -0.699 | -0.240 | -0.360 | 0.121 |
| PWY0.1479 | -1.196 | 0.401 | 0.664 | 1.597 | 1.860 | -0.263 |
| PWY.6630 | -0.510 | 0.157 | 0.349 | 0.667 | 0.859 | -0.192 |
| PWY.6628 | -0.511 | 0.160 | 0.346 | 0.671 | 0.857 | -0.186 |
| PWY.5484 | 0.028 | -0.568 | -0.281 | -0.596 | -0.309 | -0.287 |
| PWY490.3 | 0.180 | -0.750 | -1.122 | -0.929 | -1.302 | 0.372 |
| PWY.5100 | 0.119 | -0.521 | -0.211 | -0.640 | -0.330 | -0.310 |
| PWY.5347 | -0.467 | 0.067 | 0.327 | 0.534 | 0.794 | -0.260 |
| PWY.7184 | -0.123 | -0.563 | -0.396 | -0.440 | -0.273 | -0.167 |
| HEMESYN2.PWY | 0.016 | -0.775 | -0.224 | -0.791 | -0.240 | -0.551 |
| PWY.6608 | 0.388 | 0.080 | -0.297 | -0.308 | -0.685 | 0.377 |
| PWY0.1297 | 0.056 | -0.514 | -0.246 | -0.571 | -0.302 | -0.268 |
| PWY.6519 | -1.045 | -0.076 | 0.249 | 0.969 | 1.294 | -0.325 |
| BIOTIN.BIOSYNTHESIS.PWY | -0.951 | -0.013 | 0.267 | 0.938 | 1.218 | -0.280 |
| PENTOSE.P.PWY | -0.066 | -0.639 | -0.305 | -0.573 | -0.239 | -0.334 |
| ANAEROFRUCAT.PWY | 0.034 | -0.498 | -0.196 | -0.533 | -0.231 | -0.302 |
| PWY.5384 | -0.421 | -0.517 | -0.051 | -0.095 | 0.370 | -0.465 |
| PWY.6700 | -0.261 | 0.235 | 0.473 | 0.496 | 0.734 | -0.237 |
| PWY.6125 | -0.090 | -0.519 | -0.325 | -0.429 | -0.235 | -0.194 |
