# Supplementary material for: Differential growth enhancement followed by notable microbiota modulation in growing-finishing pigs by Bacillus subtilis strains ps4060, ps4100, and a 50:50 strain mixture
Source: PLoS One. 2024 Sep 9;19(9):e0306014. doi: 10.1371/journal.pone.0306014 (PMC11383229; doi:10.1371/journal.pone.0306014)
Supplement: Attachment — Submitted filename: Response to Reviewers_Revision.docx [file pone.0306014.s007.docx]

**Responses to Reviewers**

The followings are our responses to the reviewers’ comments on "Differential growth enhancement followed by notable microbiota modulation in growing-finishing pigs by *Bacillus subtilis* strains ps4060, ps4100, and a 50:50 strain mixture.” We have addressed each point raised by the editor and a reviewer in a point-to-point manner. The feedback allowed us to present a more accurate account of our study.

1. PLOS One style: The style was checked and corrected accordingly following the instructions.
2. Funding Statement: This work was partly supported by the Platform Technology Development Program (RS-2022-TI023566, S3309275) and Technology Development (R&D) Support Program (S3374815) of the Ministry of SMEs and Startups (MSS, Korea) to YC. The funders had no role in study design, data collection and analysis, decision to publish, or preparation of the manuscript. There was no additional external funding received for this study.
3. Acknowledgement: We removed the funding statement from the acknowledgement section.
4. Competing Interests: YC owns over 70% stocks of a startup company, ProxEnrem INC. This manuscript could facilitate the process of registering the strains described in the manuscript as animal diet supplement in the Republic of Korea. This does not alter our adherence to PLOS ONE policies on sharing data and materials.
5. Nonauthor contact information: All raw data such as raw body weight and feed amounts, will be available upon request to either of the corresponding authors, YC or IHK, or the Dankook Smart Animal Bioresearch Center (http://www.dankook.ac.kr/web/ins403, email: 12131722@dankook.ac.kr) as indicated in the manuscript.
6. References were meticulously checked.
7. Lack of clear hypothesis: To explicitly describe testing hypothesis, we revised the last section of the last paragraph in the introduction section. “We hypothesized that (1) each B. subtilis strain (ps4100 and ps4060) would have distinct effects on the productivity and gut microbiota of finishing pigs, and (2) the enhanced productivity observed would be associated with specific modulations in the gut microbiota, including changes in microbial diversity and the relative abundance of pathogenic and beneficial bacteria. We tested whether enhanced productivity is associated with modulation of the fecal microbiota and the differential abundance of metabolic pathways predicted from the microbial community.”
